# Butyrate-Producing Probiotics Reduce Nonalcoholic Fatty Liver Disease Progression in Rats: New Insight into the Probiotics for the Gut-Liver Axis

**DOI:** 10.1371/journal.pone.0063388

**Published:** 2013-05-16

**Authors:** Hitoshi Endo, Maki Niioka, Noriko Kobayashi, Mamoru Tanaka, Tetsu Watanabe

**Affiliations:** 1 Center for Molecular Prevention and Environmental Medicine, Department of Community Health, Tokai University School of Medicine, Isehara, Japan; 2 Teaching and Research Support Center, Department of Cell Biology and Histology, Tokai University School of Medicine, Isehara, Japan; 3 Miyarisan Pharmaceutical Co. Research Laboratory, Nagano, Japan; Boston University School of Medicine, United States of America

## Abstract

Nonalcoholic fatty liver disease (NAFLD) includes simple steatosis, nonalcoholic steatohepatitis (NASH), fibrosis, cirrhosis, and hepatocellular carcinoma. The gut-derived endotoxin plays an essential role in the pathophysiological development and progression of NAFLD. By using rat models of choline-deficient/L-amino acid-defined (CDAA)-diet-induced NAFLD, we examined whether MIYAIRI 588–a butyrate-producing probiotic – prevents the progression of pathophysiological changes from steatosis to hepatocarcinogenesis. *In vivo* experiments showed that treatment with MIYAIRI 588 reduced CDAA-diet-induced hepatic lipid deposition and significantly improved the triglyceride content, insulin resistance, serum endotoxin levels, and hepatic inflammatory indexes. We also found that MIYAIRI 588 substantially increased the activation of hepatic adenosine 5′-monophosphate-activated protein kinase (AMPK) and AKT and the expression of lipogenesis- or lipolysis-related proteins. MIYAIRI 588 also improved CDAA-diet-induced delocalization and substantially decreased the expression of the tight-junction proteins intestinal zonula occluden-1 and occludin in CDAA-diet-fed rats. Further, the MIYAIRI 588-treated rats also showed remarkable induction of nuclear factor erythoid 2-related factor 2 (Nrf2) and its targeted antioxidative enzymes, which suppressed hepatic oxidative stress. *In vitro* studies revealed that treatment with sodium butyrate (NaB) also activated AMPK and AKT and enhanced Nrf2 expression by precluding ubiquitination, thereby increasing the half-life of the Nrf2 protein. Pharmacological studies and siRNA knockdown experiments showed that NaB-mediated AMPK activation induced the phosphorylation and nuclear translocation of Sirtuin 1, leading to the increased assembly of mammalian TOR complex 2 and phosphorylation of AKT at Ser473 and subsequent induction of Nrf2 expression and activation. These favorable changes caused an obvious decrease in hepatic fibrous deposition, GST-P-positive foci development, and hepatocarcinogenesis. Our data clearly established that the probiotic MIYAIRI 588 has beneficial effects in the prevention of NAFLD progression.

## Introduction

Nonalcoholic fatty liver disease (NAFLD) is the most common liver disease in Japan and United States and a significant public health concern worldwide. NAFLD includes simple steatosis, nonalcoholic steatohepatitis (NASH), fibrosis, cirrhosis, and hepatocellular carcinoma (HCC) [1]. Although NAFLD is benign, recent studies indicate that around 40% of NAFLD patients progress to fibrosis and >50% develop NASH after 4–13 years [2], [3]. The complexity and chronology of pathophysiological events leading to NAFLD and NASH; reasons for occasional progression toward chronic inflammation, fibrosis, and cirrhosis; and effective therapies for NASH remain unknown. Therefore, effective interventions should be developed for NAFLD patients likely to develop NASH and HCC.

Some mechanisms mediating the pathogenesis of NASH have been discovered. NAFLD progression is known to depend on genetic and environmental cofactors [4], [5]. The latter include bacterial translocation through the intestinal wall and intestinal bacterial overgrowth. Gut microbiota generate products such as lipopolysaccharide (LPS), a cell-wall component of gram-negative bacteria, which are delivered to the liver via the portal vein [4], [6]. Endotoxin production by gut microbiota can cause inflammation in patients with obesity, diabetes, metabolic disorder, NAFLD, and NASH [4], [5]. In murine models of NAFLD, bacterial overgrowth causes compositional changes and increased intestinal permeability by reducing the expression of tight-junction (TJ) proteins [7]. Consistently, circulating endotoxin levels are elevated in most animal models of diet-induced NAFLD and NASH [8]. Additionally, plasma endotoxin levels are significantly higher in patients with NAFLD of different histological severities and associated with small intestinal bacterial overgrowth and increased intestinal permeability [9]. Furthermore, a complex mechanism involving extensive lipid accumulation, systemic inflammation, oxidative stress, and insulin resistance causes cytotoxicity and exacerbated hepatopathy [1], [5], [10]. Since obesity, NAFLD, and NASH are associated with a shift of the gut microbiota profile, treatment with probiotics to modify the intestinal flora may prevent NAFLD progression.

*Clostridium butyricum* is a butyric acid-producing gram-positive anaerobe found in the soil and in the intestines of healthy animals and humans [11]. The MIYAIRI 588 strain of *C. butyricum* is used as a probiotic for treating and preventing non-antimicrobial-induced diarrhea, antimicrobial-associated diarrhea, and constipation in humans [12], [13]. Butyrate is a short-chain fatty acid (SCFA) produced by microbiota in the colon and distal small intestine from resistant starch, dietary fiber, and low-digestible polysaccharides by fermentation [14]. Clinical trials show that luminal instillation of butyrate is a promising therapy for ulcerative colitis and related inflammatory disorders [15], [16].

Dietary supplementation of butyrate induces the activation of adenosine 5′-monophosphate-activated protein kinase (AMPK), thereby preventing and treating high-fat-diet-induced obesity and insulin resistance in mice [17]. AMPK regulates energy homeostasis via its effects on glucose and lipid metabolism [18], controls fatty acid oxidation by regulating mitochondrial biogenesis, and suppresses the lipogenic gene expression by reducing the activity of the transcriptional factors sterol-regulatory element-binding protein 1c (SREBP-1c) [19]. Hepatic AMPK also decreases hepatic lipogenesis, and AMPK activity can inhibit reactive oxidative stress (ROS) and inflammation [20]. *In vitro* experiments have shown that NaB treatment increased AMPK activity and accelerated the assembly of TJ proteins in the colonic epithelial cell line Caco-2 [21].

We hypothesized that MIYAIRI 588 may prevent NAFLD progression by improving the environment of gut microbiota and activating hepatic energy metabolism. Therefore, we investigated the effects of MIYAIRI588 on NAFLD progression in animals fed on choline-deficient/L-amino acid-defined (CDAA) diet.

## Materials and Methods

### Reagents

Materials were obtained from the following sources: sodium butyrate (NaB), human recombinant insulin, and D-glucose were purchased from Wako Pure Chemical (Osaka, Japan); 5-aminoimidazole-4-carboxamide, 1-D-ribonucleoside (AICAR), compound C, LY294002, and MG132, from Calbiochem-Merck (Darmstadt, Germany); and Dulbecco’s modified Eagle’s medium (DMEM), cycloheximide (CHX), dimethylsulfoxide (DMSO), and a proteinase inhibitor cocktail, from Sigma (St. Louis, MO, USA).

### Animals and Experimental Protocol

All animals used in the present study received humane care, and the experiment protocols were approved by the Animal Experiment Committee of Tokai University (Permit Number 114007). Male Fischer 344 rats weighing 70 g were purchased from CLEA Japan Inc. (Tokyo, Japan). After acclimatization for 1 week on a standard diet, the rats were divided into 3 groups. Group 1 received a CDAA diet to establish animal models for diet-induced liver steatosis, steatohepatitis, fibrosis, cirrhosis, and hepatocarcinogenesis. Group 2 received CDAA diet containing MIYAIRI 588, a pharmaceutical product of *C. butyricum*, (8.5 × 10^9 ^cfu/g, Miyarisan Pharmaceutical Co., Ltd., Tokyo, Japan) after feeding on the CDAA diet for 2 weeks. In a preliminary experiment and the current study, we ensured that the feeding of the CDAA diet caused liver steatosis for 2 weeks (Figure S1). Therefore, 10% of the total amount of CDAA diet was replaced with excipients containing MIYAIRI 588 at 2 weeks after the commencement of this experiment. Group 3 received a corresponding control choline-sufficient/L-amino acid-defined (CSAA) diet. The CSAA and CDAA diets were obtained in powdered form from Dyets Inc. (Bethlehem, PA, USA). In the both CSAA and CDAA group, 10% of the total amount of the diet was replaced with the same amount of excipients (placebo) only. A subgroup of each group was killed at 8, 16, and 50 weeks after the diet regimens were completed, and subjected to morphologic, biochemical, and molecular biologic analyses (Figure S1). For the all analyses, at least 6 rats per group were used in this experiment.

### Histologic Examination

Conventional histologic examination was performed by hematoxylin and eosin, Azan-Mallory, Sirius-red, and Oil-red O staining of excised liver sections, as described previously [22], [23], [24], [25].

### Immunohistochemistry

For immunostainings of α-smooth muscle actin (α-SMA), 4-hydroxy-2-nonenal (4-HNE), and glutathione S-transferase placental form (GST-P), 5-µm-thick tissue sections were stained by the indirect immunoperoxidase method with anti-α-SMA (Sigma), anti-4-HNE (JaiCA, Shizuoka, Japan), and anti-GST-P (MBL, Nagoya, Japan) antibodies as described previously [26], [27].

For the immunofluorescence examination, liver tissues were embedded in OCT compound (Sakura, Tokyo, Japan) and snap-frozen in liquid nitrogen, by using a previously described method with minor modifications [22]. To detect the expression levels in liver tissue, antibodies against Nrf2, GST-P, α-SMA, and desmin (Dako, Carpinteria, CA) were used together with the appropriate secondary antibodies conjugated with Qdot 605, Qdot 655, or AlexaFluor488 (Molecular Probes, Eugene, OR). Nuclei were stained with TOTO-3 (Molecular Probes). Fluorescent signals were observed and analyzed using the confocal laser-scanning microscope LSM 510 META (Carl Zeiss, Jena, Germany).

### Detection of Intracellular ROS Generation

For the detection of hepatic superoxide production, an oxidative fluorescent dye dihydroethidium (DHE) was used to evaluate the *in situ* production of superoxides [28], [29]. Unfixed fresh liver tissue samples were embedded in OCT compound (Sakura, Tokyo, Japan) and snap frozen in liquid nitrogen. Then, 10-µm-thick sections were incubated at 37°C for 30 min with 10 µM DHE (Molecular Probes). Fluorescent signals were obtained using a confocal laser-scanning microscope.

### Biochemical Analyses

Serum alanine aminotransferase (ALT) level was measured with the ALT colarendopoint assay kit (Bioo Scientific, Austin, TX). Total triacylglycerol content (TG) in liver tissue was determined by the colorimetric method using the triglyceride quantification kit (BioVision, Mountain View, CA). Hepatic tumor necrosis factor-α (TNF-α) level was measured by ELISA using the Quantikine Rat ELISA kit (R&D Systems, Minneapolis, MN, USA). The degree of lipid peroxidation in the liver was assessed by measurement of the malondialdehyde (MDA) levels using the TBARS Assay Kit (Cayman Chemical, MI, USA).

### Assessment of Insulin Resistance

Serum insulin level was measured by rat insulin ELISA kit (Sibayagi, Gunma, Japan). Peripheral blood glucose concentration was determined using an automated glycemia reader (Glutest sensor and Glutest Every; Sanwa Kagaku Kenkyusho, Nagoya, Japan). Homeostasis model assessment of insulin resistance (HOMA-IR) was calculated using the following formula: [immunoreactive insulin (µU/mL) × fasting blood sugar (mg/dL) ÷ 405] [30].

### Endotoxin Assay

Blood samples were collected from the portal vein. Endotoxin plasma levels were determined using the commercially available Pyrochrome LAL kit (Associates of Cape Cod, Falmouth, MA) according to the manufacturer’s instructions.

### Cell Culture and Treatment

Cells of the human hepatoma cell line HepG2 (Japanese Cancer Research Resources Bank, Osaka, Japan) were cultured in DMEM supplemented with 10% fetal bovine serum, 50 U/mL penicillin, 50 µg/mL streptomycin, and non-essential amino acids (Gibco BRL, Paisley, UK). To establish an *in vitro* model of insulin resistance, HepG2 cells were incubated in serum-free DMEM containing either normal concentrations of glucose (5.5 mM D-glucose) or high concentrations of glucose (30 mM D-glucose) for 24 h, as described elsewhere [31], [32]. Cells were treated with 100 nM insulin for 20 min prior to harvest.

### RNA Interference and Transfection

Gene expression was silenced using the FlexiTube GeneSolution small interfering RNAs (siRNAs) targeting *PRKAA1* (*AMPKa1*), *SIRT1*, *RICTOR*, and *AKT1* (Qiagen Valencia, CA). AllStars negative-control siRNAs was used as the negative control (Qiagen). Transfection of cells with 50 nM siRNA was performed using a Hiperfect transfection reagent (Qiagen) according to the manufacturer’s instructions.

### Protein Isolation and Western Blotting

Preparation of total, cytosol, or nucleus protein extracts from cells and tissues; electrophoresis; and subsequent blotting were performed as described previously [22], [33]. Membranes were probed with the antibodies against α-SMA, α-tubulin, β-actin (Sigma), Nrf2, PPAR-α, PPAR-γ, SREBP-1c, Lamin B (Santa Cruz Biotechnology, Santa Cruz, CA), AKT, phospho-AKT (Ser473), phospho-AKT (Thr308), AMPK-α, phospho-AMPK (Thr172), mTOR, rictor, raptor, ubiquitin, p65 NF-κB (Cell Signaling, Beverly, MA), collagen I, UCP-2, TRX (Abcam, Cambridge, UK), Keap1, NQO1, SIRT1 (Proteintech group, Chicago, IL), ZO-1, Occludin (Zymed Laboratories, San Francisco, CA), BSEP (Kamiya Biomedical, Seattle, WA), HO-1 (Enzo Life Sciences, Plymouth Meeting, PA), histone H3 (Millipore, Bedford, MA), and GST-P (MBL). For the detection of human Nrf2, SIRT1, and phosphorylated SIRT1 (Ser47), specific antibodies anti-Nrf2 (Abcam), anti-SIRT1 (Millipore), and phospho-SIRT1 (Cell Signaling), respectively, were used. Collagen I was detected using the non-denaturing protocol.

### Immunoprecipitation

Immunoprecipitation was performed using the Pierce Crosslink Immunoprecipitation Kit (Thermo Scientific, Rockford, IL) according to the manufacturer’s instructions. For immunoprecipitation of endogenous mTORC1 and mTORC2, complexes from total cell lysates were prepared in CHAPS-containing lysis buffer (40 mM HEPES of pH 7.4, 150 mM NaCl, 1 mM EDTA, 10 mM 2-glycerophosphate, 0.3% CHAPS, and proteinase inhibitor cocktail [Sigma]) were carried out as described previously [34], [35], [36]. For the detection of Nrf2 and ubiquitin complexes, cells were lysed and washed by RIPA buffer (Sigma). Immunoprecipitated proteins were then denatured and separated from the sepharose beads by adding SDS-sample buffer and boiling for 5 min, resolved by SDS-PAGE, and analyzed by western blotting.

### CHX Chase Experiment

Post-transcriptional regulation of both the steady-state levels and half-life of Nrf2 protein was investigated by CHX chase analysis as described previously [33].

### Statistical Analysis

Data were expressed as the mean ± SD. Analysis of variance was used to compare the differences between the groups. Differences were analyzed by Student’s *t* test. A *P* value of <0.05 was considered statistically significant.

## Results

### MIYAIRI 588 Treatment Prevents NAFLD Progression

**Figure 1:**
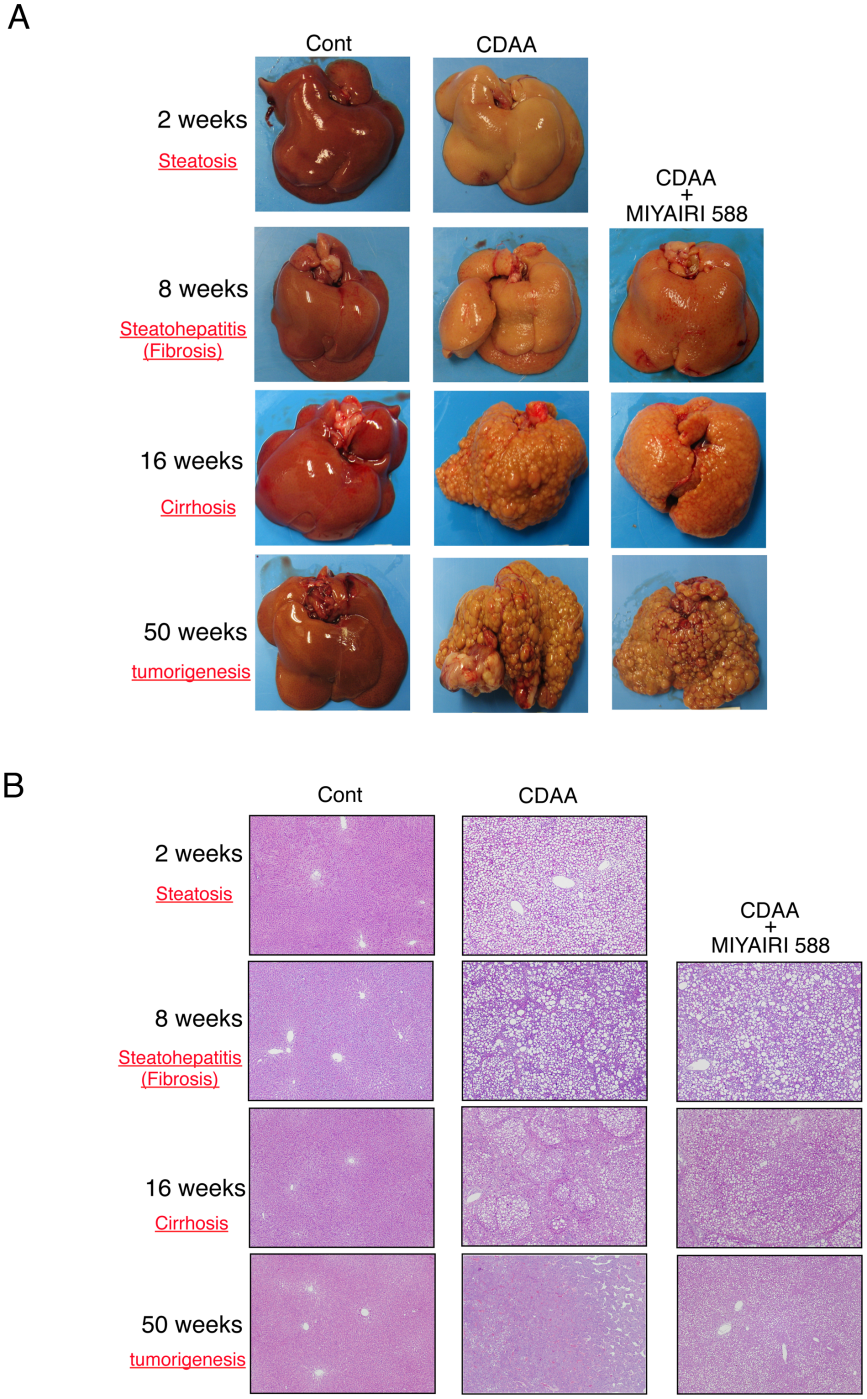
MIYAIRI 588 prevents progression of CDAA diet-induced liver steatosis to tumorigenesis. Each group was investigated at 8, 16, and 50 weeks after completion of the diet regimen. MIYAIRI 588 was administered after CDAA diet feeding for 2 weeks. To confirm that the feeding of the CDAA diet caused the liver steatosis, the groups administered CSAA and CDAA were examined at 2 weeks after the commencement of this experiment. (A) Macroscopic histomorphology and (B) hematoxylin and eosin staining for microscopic histopathology were performed at the indicated time periods. Data are representative of 6 individual liver sections. Original magnification, ×25.

Histologically, the livers of rats fed the CDAA diet for 2, 8, 16, and 50 weeks showed severe steatosis, fibrosis, cirrhosis, and tumorigenesis, respectively, implying that CDAA diet feeding induced NAFLD, NASH, liver cirrhosis, and eventually, tumor development (Figure 1). The CSAA-diet-fed control rats showed no macroscopic or histologic changes, while the coadministration of MIYAIRI 588 and CDAA diet caused remarkable amelioration of hepatic gross appearance. These results suggested that treatment with MIYAIRI 588 delays the CDAA-induced NAFLD progression and ultimately liver tumorigenesis. Significant differences between the body weights of the rats in the CDAA-diet and MIYAIRI 588-fed groups were observed at 4 and 8 weeks (Figure S2A); however, the liver-to-body weight showed no significant intergroup difference (Figure S2B).

### Reduction of Liver Steatosis and Insulin Resistance by MIYAIRI 588

**Figure 2:**
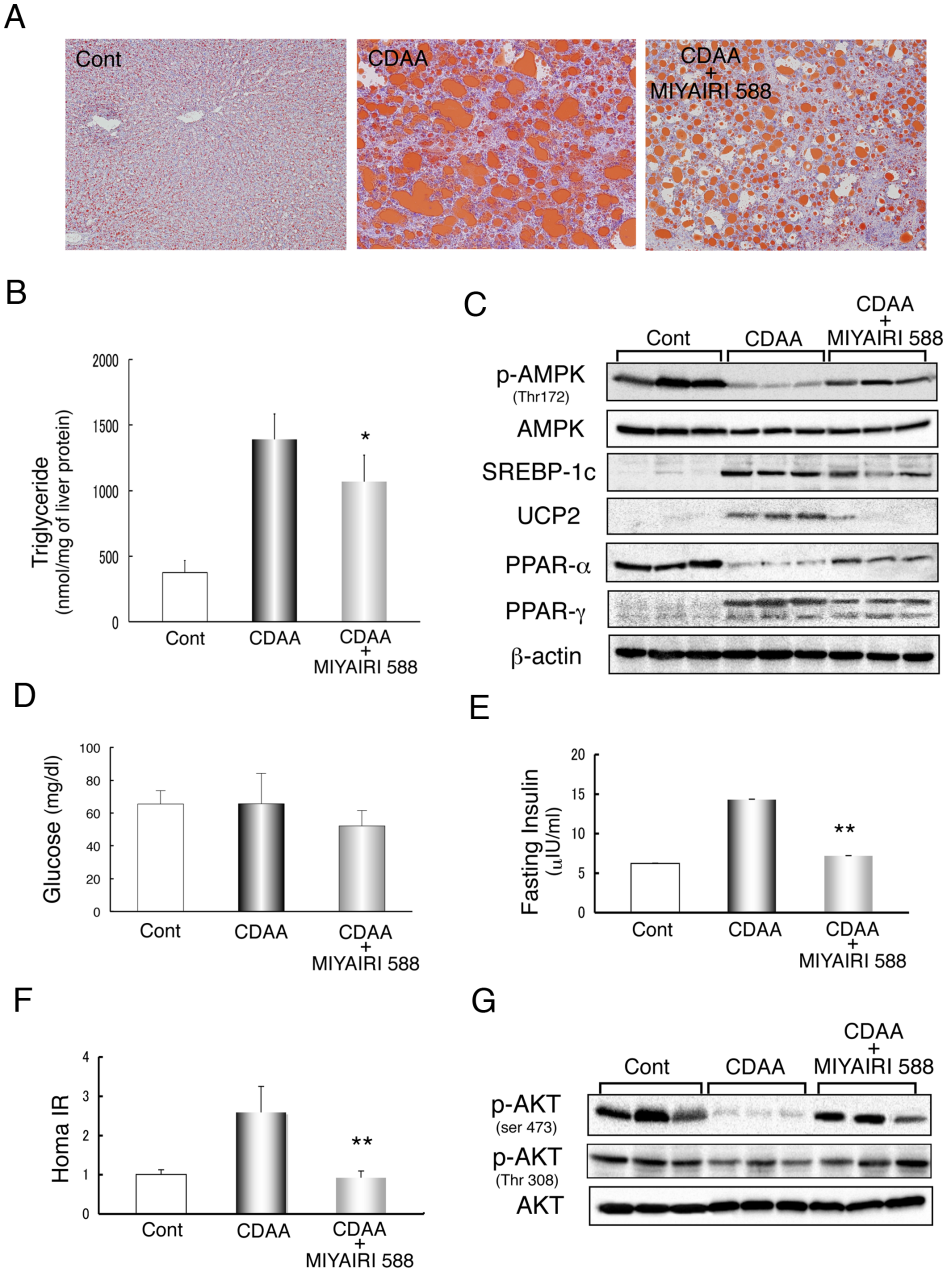
MIYAIRI 588 reduced hepatic lipid deposition and insulin resistance. Male Fischer 344 rats (n  = 6 per group) were fed a control (choline-sufficient/L-amino acid-defined CSAA) diet, choline-deficient/L-amino acid-defined (CDAA) diet, or CDAA diet plus MIYAIRI 588 for 8 weeks. MIYAIRI 588 was administered after CDAA diet feeding for 2 weeks, as described in the Materials and Methods. Cont, control. (A) Lipid accumulation was evaluated by oil red O staining of the liver sections. Data are representative of 6 individual liver sections. Original magnification, ×40. (B) Total triacylglycerol (TG) content in the liver was measured and normalized to protein concentration. Results represent mean ± SD values. *p<0.05 versus the CDAA-diet-fed group. (C) AMPK activation and lipogenesis- or lipolysis-related protein expression were detected by western blotting. β-actin expression was used as a loading control. (D) Fasting blood glucose levels, (E) Fasting plasma insulin levels, and (F) HOMA-IR were assessed in the rats. The data are shown as mean ± SD values. **p<0.01 vs. the CDAA-diet-fed group. (G) Total and phosphorylated AKT (Ser473 and Thr308) were represented under regular feed conditions.

We further investigated the effects of MIYAIRI 588 on hepatic lipid deposition by oil red O staining. Hepatic lipid accumulation and total triacylglycerol (TG) content were suppressed in the MIYAIRI 588-treated rats (Figure 2A and B). To investigate whether AMPK might be responsible for the protective effects of MIYAIRI 588, hepatic AMPK activity was assessed by determining the phosphorylation state of AMPK (Figure 2C). Hepatic AMPK phosphorylation was decreased in CDAA-diet-fed rats and was remarkably improved by MIYAIRI 588. Expression of hepatic peroxisome proliferator-activated receptor (PPAR)-α–a well-characterized target of AMPK–was also observed in MIYAIRI 588-treated rats. However, the expression of sterol regulatory element binding protein (SREBP)-1c, uncoupling protein 2 (UCP2), and PPAR-γ, which are involved in hepatic lipogenesis in high-fat-diet-induced or genetically controlled obesity, was induced in CDAA-diet-fed rat livers and suppressed by MIYAIRI 588. No significant change in the total AMPK level was evident. The fasting glucose levels were similar in the 3 groups (Figure 2D). The fasting plasma insulin levels and the calculated HOMA-IR were dramatically reduced by MIYAIRI 588 (Figure 2E and F). Further, disturbances in hepatic AKT phosphorylation at Ser473 in CDAA-diet-fed rats under feeding conditions were considerably reduced in MIYAIRI 588-treated rats, while AKT phosphorylation at Thr308 and the total AKT level remained unchanged (Figure 2G).

### MIYAIRI 588 Suppresses Endotoxin Levels and Improves Gut Permeability

**Figure 3:**
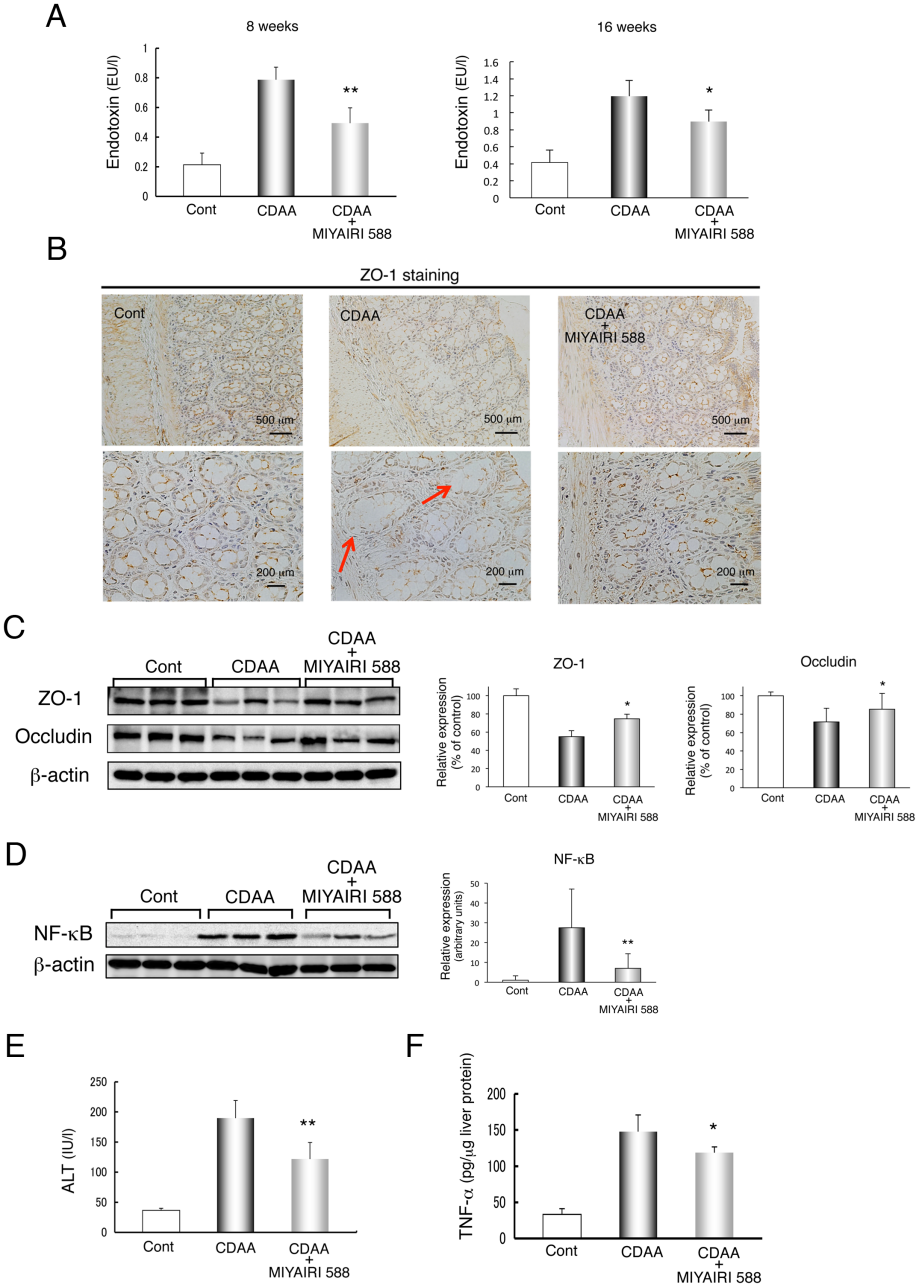
MIYAIRI 588 improves endotoxin levels in the portal vein and restores tight-junction protein expression. Rats were fed a control (CSAA) diet, CDAA diet, or CDAA diet plus MIYAIRI 588 for 8 or 16 weeks. MIYAIRI 588 was administered after 2 weeks of CDAA diet feeding. Cont, control. (A) Serum levels of endotoxin are shown. Mean ± SD values from 6 rats per group are indicated. *p<0.05, **p<0.01 compared to the CDAA-diet-fed group for the CDAA diet plus MIYAIRI 588 group. (B) The organization and distribution of tight junction proteins on intestinal tissues were examined by immunohistochemistry. Arrows indicate a disrupted intestinal barrier. Data are representative of 6 individual intestinal sections. Scale bars  = 500 µm (upper panels) or 250 µm (lower panels). (C) Expression of ZO-1 and occludin were subjected to western blot analysis in the intestinal tissues. ZO-1 and occludin expression levels were measured by densitometric analysis. β-actin expression was used as a loading control. Data are expressed as mean ± SD values. *p<0.05 compared to the CDAA-diet-fed group for the CDAA diet plus MIYAIRI 588 group. (D) The nuclear levels of the p65 subunit of NF-κB were detected by western blotting analysis of the liver samples. Expression of p65 NF-κB was normalized as a ratio to β-actin expression as a loading control. Data are expressed as mean ± SD values. **p<0.01 vs. the CDAA-diet-fed group. (E) Serum ALT levels were determined from 6 individual samples from each group. (F) Hepatic TNF-α protein level was analyzed by an enzyme-linked immunosorbent assay. Mean ± SD values for 6 rats per group are indicated. *p<0.05 compared to the CDAA-diet-fed group for the CDAA diet plus MIYAIRI 588 group.

The gut-derived endogenous endotoxin plays a critical role in the development and progression of insulin resistance, NAFLD, and NASH [5]. Serum endotoxin levels in the portal vein were higher in the CDAA-diet-fed rats than the CSAA-fed rats, but lower in the MIYAIRI 588-fed rats than the former at 8 and 16 weeks (Figure 3A). Gut permeability is controlled by TJ proteins, including zonula occluden-1 (ZO-1) and occludin. Immunohistochemical and western blot analyses showed that the strong expression and intact network of ZO-1 were predominant at crypts in intestinal sections of CSAA-fed control rats (Figure 3B and C). Importantly, the delocalization and substantial decrease in ZO-1 expression in the intestinal sections of CDAA-diet-fed rats were dramatically improved by MIYAIRI 588. Similar results were obtained on staining for occludin expression in the intestinal sections (Figure 3C and Figure S3). Thus, CDAA-diet-induced increase in hepatic NF-κB expression, plasma ALT levels, and hepatic tumor necrosis factor-α (TNF-α) production were significantly suppressed by MIYAIRI 588 treatment by decreasing endotoxin levels and enhancing TJ protein expression (Figure 3D, E, and F).

### Suppression of Hepatic Oxidative Stress and Induction of Nrf2 by MIYAIRI 588 Treatment

**Figure 4:**
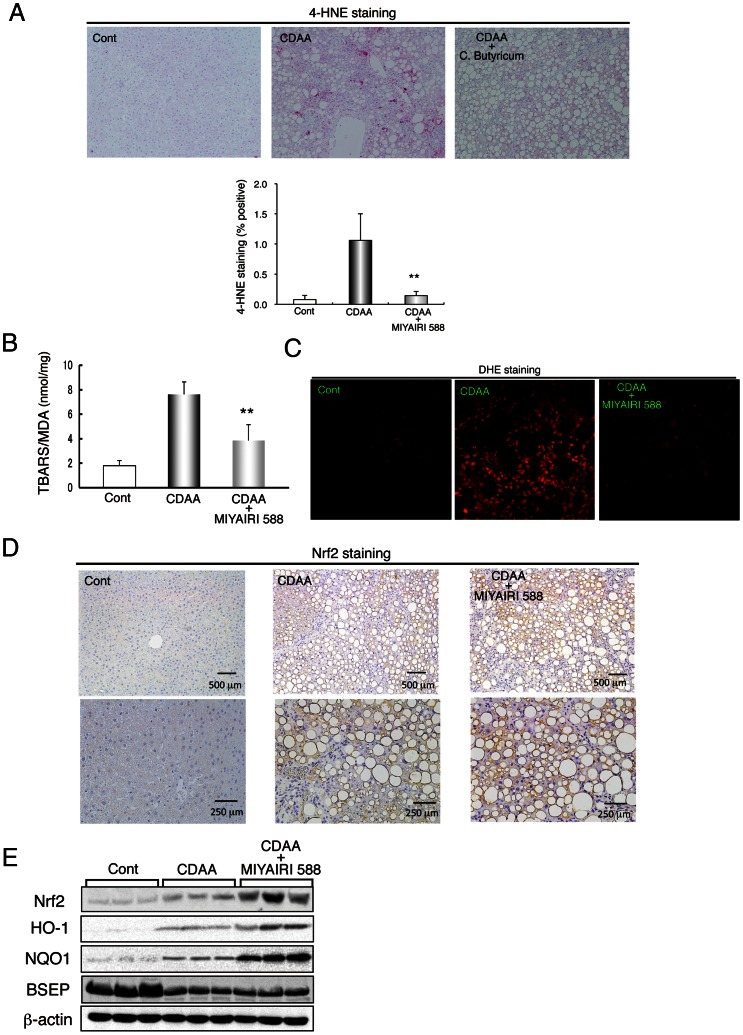
MIYAIRI 588 suppressed hepatic oxidative stresses and induced nuclear factor erythoid 2-related factor 2 expression. Rats were fed a control (CSAA) diet, CDAA diet, or CDAA diet plus MIYAIRI 588 for 8 weeks. MIYAIRI 588 was administered after CDAA diet feeding for 2 weeks. Cont, control. (A) 4-HNE-stained sections of liver specimens have been shown. The data are representative of 6 individual liver sections. Original magnification, ×40. Quantitative analysis of 4-HNE protein adducts was performed by counting the 4-HNE-positive cells for every 5 centrilobular areas of the liver tissue sections obtained for each group (lower panel). (B) Hepatic MDA levels were measured in 3 groups. The data show mean ± SD values. **p<0.01 vs. the CDAA-diet-fed group. (C) Hepatic superoxide generation was detected with dihydroethidium staining in 3 groups. (D) Immunostaining of Nrf2 was shown in the liver of the rats of each group. Data are representative of 6 individual liver sections. Original magnification, ×40 (upper panels) or ×100 (lower panels). (E) Expression of Nrf2 and its targeted genes encoding enzymes, such as HO-1, NQO1, and BSEP, was examined by western blot analysis in the liver samples of each group. β-actin expression was analyzed as a loading control.

Oxidative stress and the release of ROS probably contribute to the development of NASH, which is further complicated by the elevation of intestinal endotoxin that induces ROS production in the liver [5]. Therefore, we first measured the hepatic levels of the lipid peroxidation products 4-hydroxynonenal (4-HNE) and malondialdehyde (MDA), as indicators of oxidative stress, after 8 weeks of the diet. Immunohistochemical staining showed lower hepatic 4-HNE levels in the MIYAIRI 588-treated rats than in the CDAA-diet-fed rats (Figure 4A). Measurement of MDA using thiobarbituric acid reactive substance assay also showed that hepatic lipid peroxidation levels were lower in MIYAIRI 588-fed rats than CDAA-diet-fed rats (Figure 4B). Furthermore, DHE staining revealed that hepatic superoxide generation was much higher in the CDAA-diet-fed rats than the CSAA-fed rats, whereas hepatic DHE-positive signals were markedly decreased in the MIYAIRI 588-treated rats (Figure 4C).

Nrf2 is a potent transcriptional activator and is important for inducing of the expression of many cytoprotective genes triggered in response to oxidative stresses [37]. Immunostaining analysis showed that induction of hepatic Nrf2 expression at 8 weeks and expression of the Nrf2-regulated proteins heme oxygenase-1 (HO-1) and NAD(P)H:quinone oxidoreductase 1 (NQO1) were greater in the MIYAIRI 588-treated rats than CDAA-diet-fed rats (Figure 4D and E). Nrf2 expression was most predominant at 16 weeks (Figure S4). Levels of bile salt export pump (BSEP), a major determinant of bile-salt-dependent bile secretion that is induced by oltipraz–an Nrf2 activator [38]–were similar in the CDAA-diet-fed and MIYAIRI 588-treated rats (Figure 4E).

#### NaB induces Nrf2 expression by stabilization of Nrf2 protein

**Figure 5:**
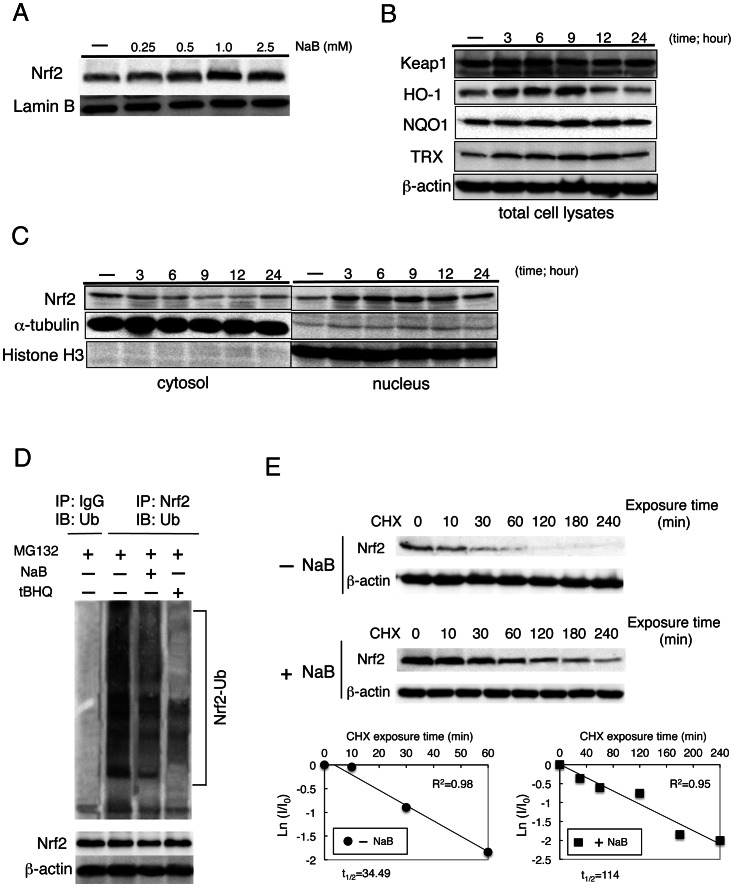
Sodium butyrate (NaB) induced Nrf2 expression and nuclear accumulation. (A) Dose-dependent effects of NaB on the expression of Nrf2 protein in HepG2 cells maintained in serum-free medium for 24 h and then treated with NaB at described concentrations for 6 h. Lamin B expression was used as a loading control. (B) Expression of Keap1, HO-1, NQO1, and TRX was examined by western blot analysis at 6 h. HepG2 cells were treated with 1.5 mM NaB at the indicated time points. (C) Nuclear accumulation of Nrf2 was stimulated by 1.5-mM NaB treatment of cells for the indicated time. Cytoplasmic and nucleic extracts were prepared and subjected to western blot analysis. Anti-α-tubulin and anti-histone H3 antibodies were used as markers for the cytoplasmic and nuclear extracts, respectively. (D) Ubiquitination of endogenous Nrf2 was assessed in HepG2 cells treated with DMSO, 1.5 mM NaB or 100 µM tBHQ for 9 h, along with 10 µM MG132. Nrf2 was immunoprecipitated with an anti-Nrf2 antibody and ubiquitinated Nrf2 was detected with an anti-ubiquitin antibody. (E) Post-transcriptional regulation of both the steady-state level and half-life of Nrf2 protein was evaluated. CHX (100 µM) was added to block protein synthesis. Cells were lysed at the indicated time points, and cell lysates were subjected to western blot analysis with anti-Nrf2 and anti-β-actin antibodies (upper panels). Lower panels depict the natural logarithm of the relative levels of the Nrf2 protein as a function of CHX chase time in the absence or presence of 1.5 mM NaB. The protein half-life has been determined in the linear range of the degradation curve.

MIYAIRI 588 induced Nrf2 expression both in the liver and intestinal tissues (Figure 4D, E, and Figure S4, S5). We further examined whether NaB directly induces Nrf2 expression, by treating HepG2 cells with NaB for different durations and doses. The increased expression of Nrf2 was just detectable at 1–2.5 µM NaB, and time-dependent induction of the Nrf2-targeted enzymes HO-1, NQO1, and thioredoxin (TRX) was also observed (Figure 5A and B). Nrf2 activation is mainly regulated by its inhibitory anchor protein Kelch-like ECH-associated protein-1 (Keap1). NaB caused the nuclear translocation of Nrf2 in a time-dependent manner, without any change in Keap1 expression levels (Figure 5C). To investigate the detailed mechanisms underlying the induction of Nrf2 expression by NaB, we examined the ubiquitination of endogenous Nrf2 by immunoprecipitation experiments after treatment with the MG132–a 26 S proteasome inhibitor–in the presence or absence of NaB. Treatment with NaB or the Nrf2-inducer tBHQ reduced the ubiquitination of endogenous Nrf2 (Figure 5D), thereby increasing the half-life of Nrf2 from approximately 34 min to 114 min after NaB treatment (Figure 5E).

### NaB-induced Nrf2 Expression is Regulated by AMPK and AKT Activation, Sirtuin 1 Expression, and mTORC2 Modification

**Figure 6:**
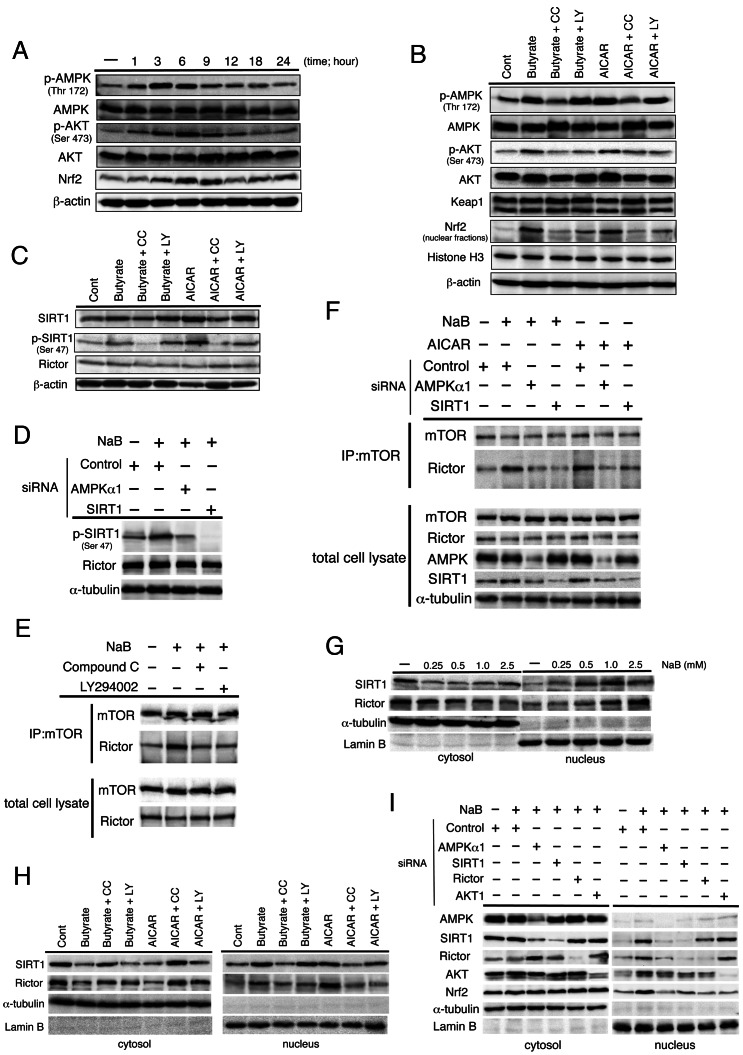
Nrf2 expression is regulated by AMPK and AKT activation and mTORC2 modification underlying NaB treatment. (A) Serum-starved HepG2 cells were treated with 1.5 mM NaB for the indicated time periods. Western blot analysis was performed with the indicated antibodies. β-actin expression was used as the loading control. (B, C) Serum-starved cells were pretreated for 1 h with AMPK agonist compound C (CC; 20 µM) or the PI3K-specific inhibitor LY294002 (LY; 25 µM) and then incubated with 1.5 mM NaB or 1 mM AMPK activator AICAR for 6 h. Western blot analysis was performed with the indicated antibodies. (D) Cells were transfected with indicated siRNAs for 48 h and then incubated with 1.5 mM NaB for 6 h under serum-starved conditions. Western blot analysis was performed with the indicated antibodies. α-tubulin expression was used as the loading control. (E) Serum-starved cells were pretreated for 1 h with 20 µM CC or 25 µM LY and then incubated with 1.5 mM NaB for 6 h. Cell lysates and mTOR immunoprecipitates (IPs) prepared from the total cell lysates were analyzed by western blotting for the levels of mTOR and rictor. (F) Cells were transfected with the indicated siRNAs for 48 h and then incubated with 1.5 mM NaB or 1 mM AICAR for 6 h under serum-starved conditions. Cell lysates and mTOR immunoprecipitates (IPs) prepared from the total cell lysates were analyzed by western blotting for the levels of mTOR and rictor. (G) Nuclear accumulation of SIRT1 or rictor was examined by western blot analysis. Cells were treated with NaB for 6 h at the indicated concentration. Anti-α-tubulin and anti-lamin B antibodies were used as markers for the cytoplasmic and nuclear extracts, respectively. (H) Serum-starved cells were pretreated for 1 h with 20 µM CC or 25 µM LY and then incubated with 1.5 mM NaB for 6 h. Nuclear accumulation of SIRT1 or rictor was examined by western blot analysis. α-tubulin and lamin B were evaluated for expression levels as markers for the cytoplasmic and nuclear extracts, respectively. (I) Cells were transfected with indicated siRNAs for 48 h and then incubated with 1.5 mM NaB for 6 h under serum-starved conditions. Western blot analysis was performed with the indicated antibodies. α-tubulin and lamin B expression were used as the loading control of cytoplasmic and nuclear extract proteins, respectively.

Next, we analyzed the correlation between activation of AMPK and AKT and expression of Nrf2 in NaB-stimulated HepG2 cells. The phosphorylation of AMPK and AKT resulted in the induction of Nrf2 expression in the cells (Figure 6A), thereby confirming our *in vivo* findings. Further, NaB-induced phosphorylation of AKT and expression of Nrf2 were blocked by the AMPK-selective antagonist compound C (Figure 6B). However, phosphatidylinositol 3-kinase (PI3K)-specific inhibitor LY294002 did not inhibit NaB-induced AMPK phosphorylation but partially suppressed AKT phosphorylation and Nrf2 expression. Keap1 expression remained unchanged. Similar results were obtained when another AMPK-specific agonist, 5-amino 4-imidazolecarboxamide riboside (AICAR), was used. AMPK regulates Sirtuin 1 (SIRT1) activity in some tissues [39] and SIRT1 promotes the expression of the mammalian TOR complex 2 (mTORC2) component rictor in the liver [40]. mTORC2 specifically phosphorylates AKT at Ser473 [41]. Our *in vivo* experiments showed that hepatic SIRT1 expression was reduced in CDAA-diet-fed rats and remarkably improved by MIYAIRI 588 under feeding conditions. MIYAIRI 588-mediated SIRT1 induction was detected more clearly after fasting (Figure S6). We also found that NaB or AICAR treatment induced SIRT1 expression only marginally, but SIRT1 phosphorylation clearly occurred at Ser47 and was completely inhibited by compound C, but not by LY294002 (Figure 6C). No significant change was detected in rictor expression. Further, NaB-induced phosphorylation of SIRT1 was abolished by AMPKα1 siRNA, while the expression of rictor was not affected by AMPKα1 or SIRT1 siRNA (Figure 6D). Next, we determined whether the formation of mTORC2 components was affected by NaB. The coimmunoprecipitation assay results showed an increased assembly of endogenous rictor and mTOR, which was reduced by compound C or LY294002 treatment (Figure 6E). In addition, the rictor and mTOR association augmented by NaB or AICAR was completely diminished by AMPKα1 or SIRT1 siRNA (Figure 6F). If the expression of SIRT1 or rictor caused an alteration in the signal transduction by NaB, we reasoned that intracellular distributions of these proteins would be accompanied by dynamic alterations in the cells. NaB or AICAR treatment were indeed found to cause a dose-dependent nuclear accumulation of SIRT1 and rictor (Figure 6G). This nuclear translocation of SIRT1 and rictor was impeded by compound C. However, LY294002 did not inhibit the nuclear accumulation of SIRT1, but not rictor (Figure 6H). We also found that inhibition of AMPKα1 was associated with diminished NaB-induced nuclear accumulation of SIRT1, rictor, AKT, and Nrf2 in the cells. SIRT1 siRNA impaired NaB-mediated translocation of rictor into the nucleus, AKT, and Nrf2. Similarly, siRNA targeting rictor blocked the NaB-induced nuclear transport of AKT and Nrf2 from the cytoplasm. A siRNA-mediated knockdown of AKT1 impaired only the nuclear accumulation of Nrf2 underlying NaB treatment without affecting the expression of AMPK, SIRT1, and rictor (Figure 6I).

### NaB Overcomes Signal Transduction Deteriorated by Insulin Resistance

**Figure 7:**
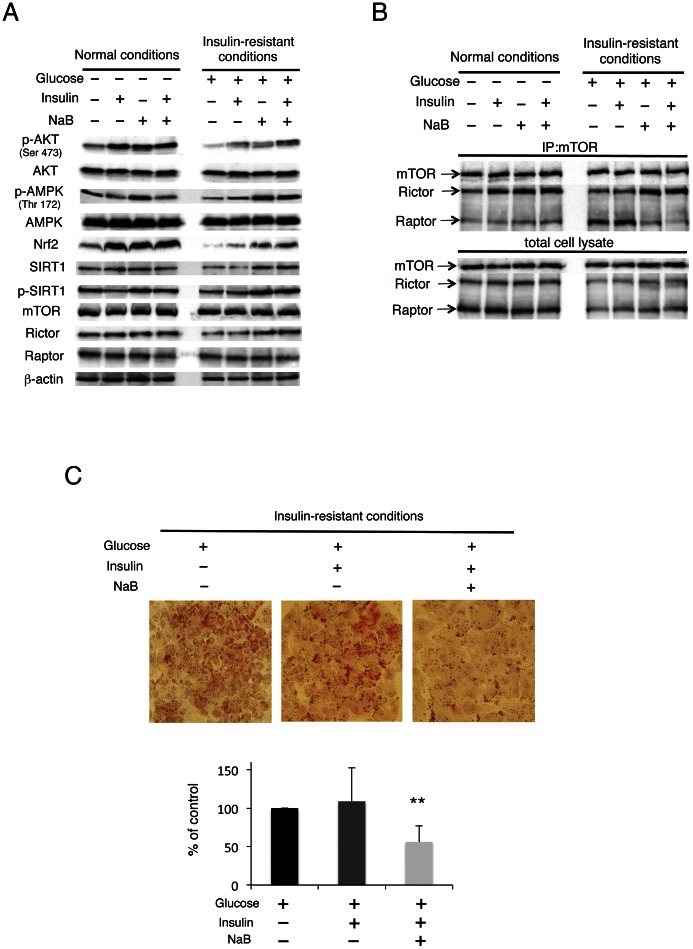
NaB improves insulin signaling and insulin-resistance conditions. (A) Serum-starved cells were incubated in a medium containing either normal or high glucose concentration for 24 h and then treated with or without 1.5 mM NaB for 6 h. Subsequently, the treated cells were stimulated with 100 nM insulin for 20 min. Western blot analysis was performed with the indicated antibodies. (B) Normal or insulin-resistance conditions were established, and cell lysates and mTOR immunoprecipitates (IP) prepared from the total cell lysates were analyzed by immunoblotting for the levels of mTOR, rictor, and raptor. (C) Lipid accumulation was examined by oil-red O staining in insulin-resistance conditions. Serum-starved cells were incubated in a medium containing high glucose concentration for 24 h and then treated with or without 1.5 mM NaB for 9 h in the absence and presence of insulin. Quantification of the extent of oil-red O staining was done by counting red-stained regions for every 3 centrilobular areas of the cells in each group (lower panel). The values represent the mean ± SD values. **p<0.01 compared with the group exposed to high glucose levels in the absence insulin and NaB.

We created a model of insulin resistance by exposing HepG2 cells to high concentrations of glucose and examined the effect of NaB under insulin-resistance conditions. Indeed, insulin-triggered AKT phosphorylation was almost completely abolished under these conditions (Figure 7A), while phosphorylation of AMPK and SIRT1 and expression of Nrf2 and SIRT1 were reduced; these reductions were markedly improved by NaB with or without insulin. However, the expression levels of mTOR, rictor, and a mammalian TOR complex 1 (mTORC1) component, raptor, remained unaltered. Since the binding of rictor to mTOR was increased by NaB under normal conditions (Figure 6E), we hypothesized that AKT activation would be recovered by NaB treatment through the augmentation of mTORC2 assembly under insulin-resistance conditions. Endogenous rictor–mTOR interaction was enhanced, whereas the raptor–mTOR complex was unaffected by stimulation with insulin and/or NaB, under normal conditions (Figure 7B). Conversely, decreased rictor–mTOR and increased raptor–mTOR interaction were noted under insulin-resistance conditions. Treatment with insulin and/or NaB did not alter the raptor–mTOR interaction under normal conditions. While the formation of these complexes was augmented by insulin-resistance condition, the raptor–mTOR interaction was largely abolished by NaB treatment. Oil-red O staining showed that increased lipid accumulation induced by high glucose was significantly reduced by NaB treatment with insulin in HepG2 cells (Figure 7C).

### MIYAIRI 588 Prevents CDAA-diet-induced Liver Fibrosis and Hepatocarcinogenesis in Rat Liver

**Figure 8:**
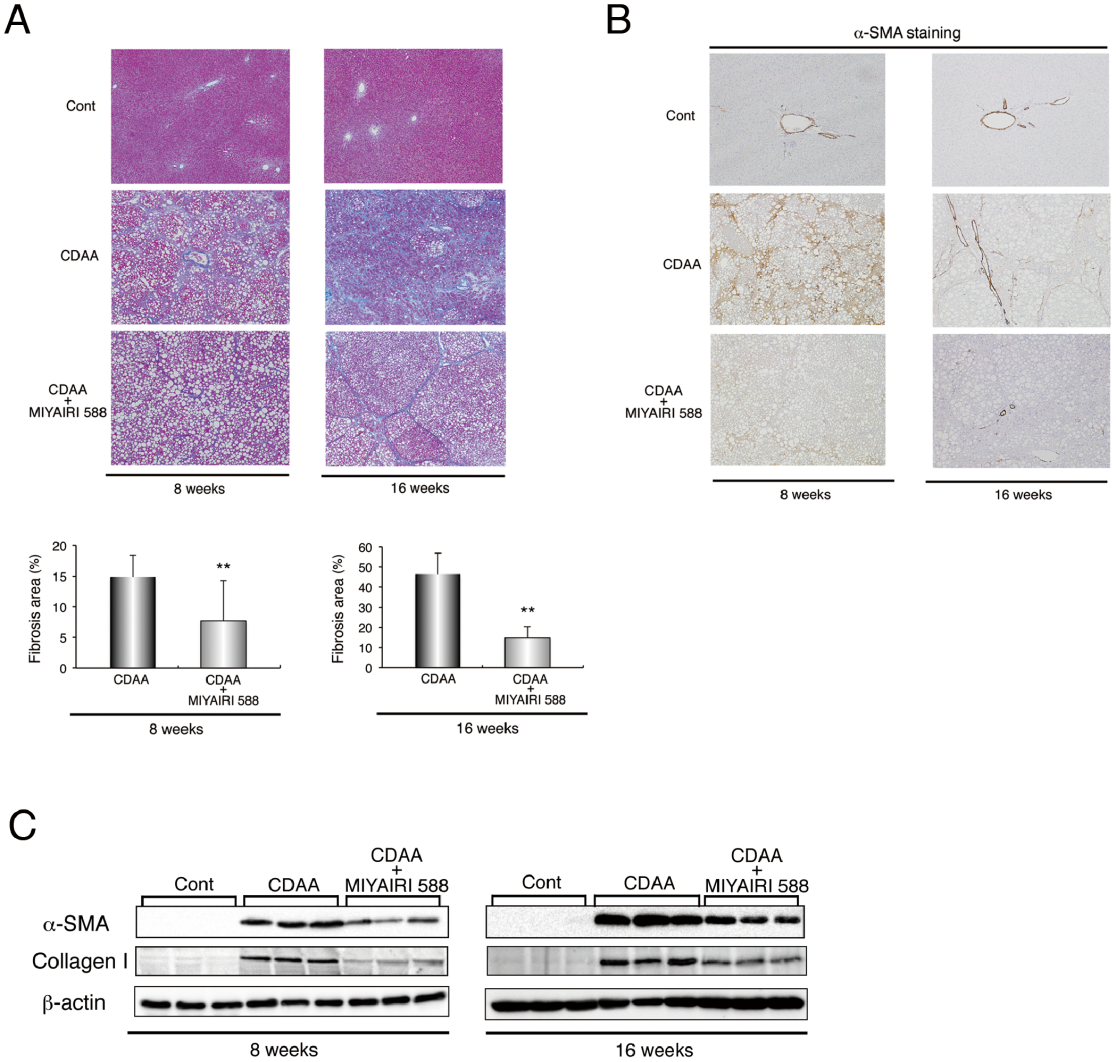
MIYAIRI 588 prevents the progression of CDAA-diet-induced liver fibrosis and cirrhosis. Male Fischer 344 rats (n  = 6 per group) were fed a CSAA diet (Cont), CDAA diet, or CDAA diet plus MIYAIRI 588 for 8 or 16 weeks. MIYAIRI 588 was administered after CDAA diet feeding for 2 weeks. (A) Hepatic fibrosis was assessed by Azan-Mallory staining. Data are representative of 6 individual liver sections. Original magnification, ×40. The fibrosis area was assessed using image analysis techniques to calculate the ratio of connective tissue to the whole area of liver sections stained with Azan-Mallory. The data are expressed as mean ± SD values. ** p<0.01 compared with the CDAA-diet-fed group. (B) Immunostaining of α-SMA expression is shown. Data are representative of 6 individual liver sections. Original magnification, ×40. (C) α-SMA and collagen I expression was examined by western blot analysis at the indicated time points. β-actin expression was used as the loading control.

**Figure 9:**
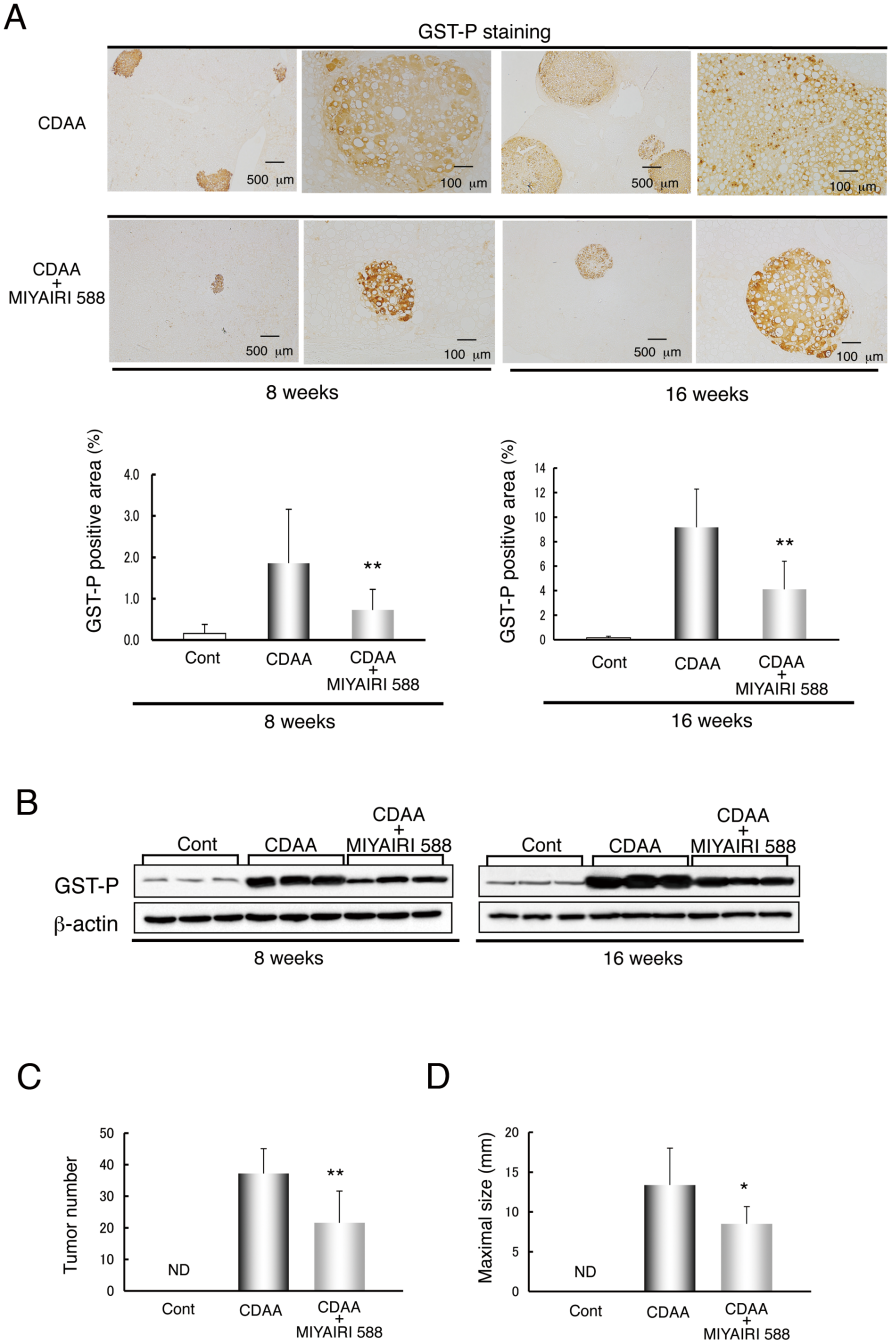
MIYAIRI 588 inhibits development of CDAA-diet-induced hepatocarcinogenesis. Rats were fed a CSAA diet (Cont), CDAA diet, or CDAA diet plus MIYAIRI 588 for 8 or 16weeks. MIYAIRI 588 was administered after CDAA diet feeding for 2 weeks. (A) Representative GST-P-positive preneoplastic foci in the liver of rats were shown. The GST-P-positive area was assessed by calculating the ratio of GST-P foci to the whole area of liver sections (lower panels). Data are representative of 6 individual liver sections. The data are expressed as mean ± SD values. Scale bars were indicated in each photograph. **p<0.01 compared with the CDAA-diet-fed group. (B) GST-P expression was detected by western blot analysis at the indicated time points. β-actin expression was used as the loading control. (C, D) Tumor number (≥1.0 mm) and maximal tumor size (diameter in mm) in the livers of rats fed on a CDAA diet (n  = 8) and CDAA diet plus MIYAIRI 588 (n  = 9) rats. Cont, control. ND, not detected. The data are expressed as mean ± SD values. *p<0.05, **p<0.01 compared with the CDAA-diet-fed group.

We examined the histologic changes in the fibrotic or cirrhotic liver at 8 and 16 weeks. Azan-Mallory staining showed an obvious decrease in fibrous deposition areas in MIYAIRI 588-treated rats (Figure 8A), as confirmed by densitometric analysis. Similarly, the hepatic protein levels of fibrosis-related factors, including α-SMA and collagen I, and the hepatic collagen deposition were lower in the MIYAIRI 588-fed rats than the CDAA-diet-fed rats (Figures 8B and C and S7). Lastly, we first investigated the hepatic expression levels of glutathione S-transferase placental form (GST-P), a marker for cellular alteration in the early stage of hepatocarcinogenesis, at 8 or 16 weeks. The area, number, and expression levels of hepatic GST-P foci were lower in the MIYAIRI 588-treated rats than in the CDAA-diet-fed rats (Figure 9A and B). Notably, CDAA diet-induced multiplicity of liver tumors was decreased. Although all the CDAA diet-fed rats developed liver tumors at 50 weeks, the number of detectable tumors (≥1.0 mm) and maximal diameters of tumors were significantly lower in the MIYAIRI 588-treated rats (Figure 9C and D).

## Discussion

This study revealed that treatment with the *C. butyricum* strain MIYAIRI 588–a butyrate-producing probiotic–prevents the progression of CDAA-induced NAFLD to liver carcinogenesis in animals. Furthermore, we found that NaB activates the AMPK/SIRT1/PI3K/mTORC2/AKT/Nrf2 signaling pathway. Thus, our results indicate an important and novel use of MIYAIRI 588 and explain the role of the gut–liver axis in nutrient-induced NAFLD.

Gut bacteria contribute to the pathogenesis of NAFLD. Leptin-deficient *ob*/*ob* and hyperleptinemic *db*/*db* mice have higher endotoxin levels in the portal blood than wild-type animals [7]. Metnionine-choline-deficient (MCD) diet and fructose intake caused NAFLD in mice, resulting in increased gut-derived endotoxin levels in portal blood [42], [43], [44]. Further, the leakage of gut-microbiota-derived LPS into the portal blood is a well-established mechanism of metabolic endotoxemia that triggers systemic inflammation and hepatic oxidative stress [4], [5], [6]. Thus, proper regulation of the intestinal environment is important to prevent NAFLD progression. Although administration of the probiotic preparation VSL#3, containing freeze-dried bacteria of 8 species, attenuates MCD-diet-induced liver fibrosis in mice, it does not reduce liver steatosis, inflammation, or serum endotoxin levels [43]. MIYAIRI 588 significantly suppressed the CDAA-diet-induced increase in endotoxin levels in the portal vein and also restored the ZO-1 and occludin levels to those in the control group. This implied that MIYAIRI 588 efficiently reduced gut-derived endotoxin levels in the portal blood by altering the intestinal flora and restoring gut-barrier functions, thereby reducing the hepatic levels of inflammatory cytokine TNF-α and TNF-α-regulated transcription factor NF-κB. Peng et al. demonstrated that butyrate enhances the intestinal barrier function by facilitating the assembly and expression of ZO-1 and occludin, depending on AMPK activation [21]. Furthermore, dietary supplementation of NaB activated hepatic AMPK in mice fed on a high-fat diet [17]. Interestingly, MIYAIRI 588 treatment prevented the CDAA-diet-induced decrease in hepatic AMPK phosphorylation. NaB also activated AMPK in HepG2 cells. While butyrate is largely utilized by the colonic epithelium as an energy source, a substantial amount of butyrate transfers from the gut to the liver via the portal vein [45]. Thus, MIYAIRI 588-produced butyrate may have direct beneficial effects on the gut and liver by altering the gut environment and AMPK activation.

AMPK increases NAD^+^ levels, activates SIRT1, and induces SIRT1-dependent PGC1α deacetylation [46], [47]. SIRT1 is activated through the phosphorylation at Ser27, Ser47, and Thr530 by c-Jun n-terminal kinase activation in response to oxidative stress and is expressed by its nuclear translocation [48]. SIRT1 is also phosphorylated by cyclin-dependent kinases (cyclin B/Cdk1), which increase its activity and regulate cell-cycle progression [49]. This is the first report identifying AMPK as a kinase that functionally modifies SIRT1, increases SIRT1 phosphorylation, and alters SIRT1 translocation upon NaB treatment. Growing evidence indicates that SIRT1 overexpression and several SIRT1 activators have beneficial effects on glucose homeostasis and insulin sensitivity in obese mice models. Liver-specific deletion of the *Sirt1* gene in mice is reported to cause liver steatosis, insulin resistance, and oxidative stress in various organs [40]. Our study clearly showed that AMPK and SIRT1 positively regulate the rictor–mTOR association underlying NaB treatment, thereby triggering AKT phosphorylation at Ser473 and improving insulin signaling. However, the hepatic overexpression of a dominant negative form of raptor enhances AKT phosphorylation and restores insulin sensitivity in K/KAy mice with genetic obesity-associated insulin resistance [50]. Consistently, we found that the interaction of raptor and mTOR was enhanced, while that of rictor and mTOR was suppressed under insulin-resistant conditions; this was restored by NaB treatment, resulting in improved AKT phosphorylation. The net effect of AMPK on AKT-mediated insulin signaling is complex and involves multiple targets. Although AMPK activation enhances insulin sensitivity, the underlying mechanisms remain unclear. Indeed, AMPK activators, such as AICAR or adiponectin, enhance the effect of insulin on AKT activation [51], [52]. Further, PI3K functions downstream of AMPK to regulate AKT activity in neuronal polarization [53]. PI3K activity of muscle is reported to be diminished in mice with a muscle-specific deletion of *Sirt1* [54]. Several growth factors activate mTORC2 via PI3K, and insulin-stimulated PI3K signaling is reported to promote mTORC2–ribosome binding activity [55]. We found that PI3K inhibitor blocked NaB-augmented mTORC2 complexes, but did not inhibit the phosphorylation and nuclear accumulation of SIRT1. Therefore, we propose that the NaB signaling pathway modulates cellular energy metabolism and insulin sensitivity by AMPK activation and subsequent regulation of AKT phosphorylation through the modification of SIRT1, PI3K, and mTORC1/2.

ROS or lipid peroxidation products activate the hepatic stellate cells (HSCs) and stimulate the production of extracellular matrix proteins, including type I collagen, in them [56]. Thus, ROS are important to the development of liver fibrosis. Nrf2 is essential for cellular protective mechanisms against ROS, electrophiles, or oxidative stresses-induced inflammation through the transcriptional activation of antioxidant-responsive element (ARE)-dependent expressions of genes encoding enzymes such as NQO1, HO-1, GSTs, and TRX [37]. Studies have indicated that high-fat or MCD diets resulted in more severe NAFLD/NASH in Nrf2-null mice than wild-type mice [27], [57]. Moreover, MCD diet substantially increased the expression of NF-κB p65 subunit in Nrf2-deficient mice liver [57]. Furthermore, NF-κB pathway or NF-κB p65 subunit antagonizes the activation of the Nrf2–ARE pathway [58]. This indicates that Nrf2 plays a pivotal role in protection against the development of NAFLD/NASH. Interestingly, Nrf2 expression colocalized with expression of desmin and α-SMA, which are markers for quiescent and activated HSCs and activated HSCs, respectively, in MIYAIRI 588-treated rat livers (Figure S8), thereby suggesting that MIYAIRI 588-induced Nrf2 expression directly inhibited HSC activation and liver fibrogenesis by protecting against oxidative stress. Further, MIYAIRI 588 induced marked expression of Nrf2 and Nrf2-regulated proteins HO-1 and NQO1 in the liver. Thus, the suppression of hepatic inflammation and oxidative stress is caused by both the reduction of gut-derived endotoxin levels and induction of MIYAIRI 588-mediated Nrf2 expression in the liver, which are important for preventing NAFLD progression. Interestingly, while Nrf2 colocalizes a part of GST-P expression in the liver of MIYAIRI 588-treated rats, GST-P expression was found even without Nrf2 in CDAA-diet-fed rat livers (Figure S9). Although GST-P level is markedly induced in preneoplastic foci and nodules of the liver, it is a phase II detoxification enzyme [59]. *GST-P* has been found to be a target gene of Nrf2 [60]. Thus, our findings suggested that GST-P-positive foci represent a distinct feature of the preneoplastic region of liver underlying dependent or independent on Nrf2 expression.

**Figure 10:**
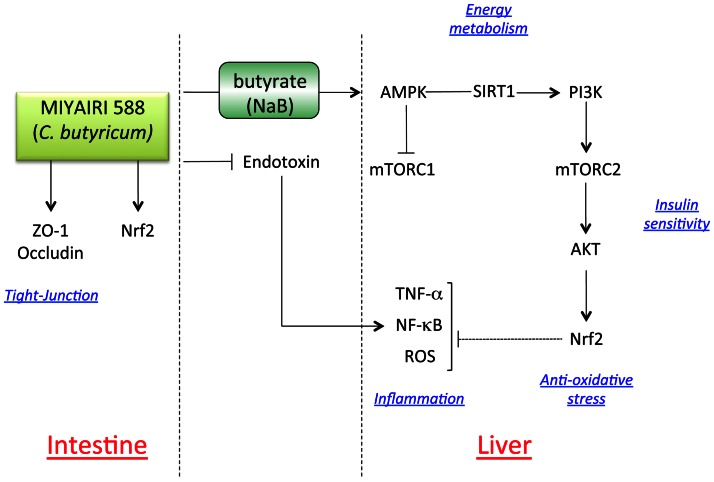
The hypothetical model of the effects of MIYAIRI 588 on the progression of NAFLD. Schematic representation of the mechanisms showing that probiotic MIYAIRI 588 prevents the progression of NAFLD through the intestine/liver axis and systematic signaling activation. MIYAIRI 588 designates the AMPK activation as a starting point and regulates different pathophysiological events, such as lipid and energy metabolism, insulin sensitivity, and oxidative stress response, tight-junction modification through consistent and systematic signaling pathways, thereby causing pronounced suppression of NAFLD progression.

Oxidative stress or chemopreventive agents disrupt the sequestration of Nrf2 by Keap1, thereby permitting the nuclear translocation of Nrf2 [37], [61]. Keap1–a substrate adaptor protein for the Cul 3-containing E3 ubiquitin ligase–negatively controls the activity of Nrf2 at the protein level by polyubiquitination and degradation, to maintain low basal levels of Nrf2 [62]. In response to chemopreventive compounds, cysteine residues in Keap1 are modified and Nrf2 is released from the Nrf2/Keap1 complex, resulting in the stabilization and activation of Nrf2. These findings are largely consistent with our results indicating that NaB stabilizes the expression of Nrf2 by facilitating the escape from ubiquitination, thereby prolonging the half-life of Nrf2 protein. Evidence suggests that more complex regulatory mechanisms may be involved in the Keap1-Nrf2-ARE machinery. Interestingly, a recent study demonstrated that AMPK induces HO-1 gene expression in vascular and arteries cells via Nrf2 induction and ARE activation [63]. PI3K-AKT pathway can also trigger the Nrf2 activation [64]. In the present study, our pharmacological investigation and siRNA knockdown experiments indicated that NaB-mediated AMPK activation induced the phosphorylation and nuclear translocation of SIRT1, thereby leading to the increasing assembly of mTORC2 and phosphorylation of AKT at Ser473, which in turn induced Nrf2 expression and activation. Consistently, our findings also indicated that MIYAIRI 588-produced NaB activates the AMPK/SIRT1/PI3K/mTORC2/AKT/Nrf2 signaling pathway in the liver. Notably, our observations revealed a new mechanism whereby treatment with MIYAIRI 588 designates the AMPK activation as a starting point and regulates different pathophysiological events, such as lipid and energy metabolism, insulin sensitivity, and oxidative stress response, through consistent and systematic signaling pathways, thereby causing pronounced suppression of NAFLD progression (Figure 10). Thus, the manipulation of gut microbiota by probiotics can help counteract the adverse effects on the liver and augment other therapeutic options for NAFLD. Although further investigations are required, we believe that our findings will facilitate the development of preventive and therapeutic interventions for NAFLD.

## Supporting Information

Figure S1 **Schematic representation of the experimental protocol.** To generate the model of nutrient-induced NASH, male Fischer 344 rats received a CDAA diet. Control group received a corresponding control CSAA diet. In the CDAA plus MIYAIRI 588 group, 10% of the total amount of CDAA diet was replaced with excipients containing MIYAIRI 588 at 2 weeks after the commencement of this experiment. In the both CSAA and CDAA group, 10% of the total amount of the diet was replaced with the same amount of excipients (placebo) only. Rats were killed on 8, 16, and 50 weeks after completion of the diet regimen.
(TIF)

Figure S2 **Effects of growth and liver weight by MIYAIRI 588.** Cont, control. (A, B) Body weight gain and liver-to-body weight were measured at indicated time points (n  = 6–10 per group). Values are expressed as means± SD. **p < 0.01 vs. the CDAA diet-fed group.
(TIF)

Figure S3 **MIYAIRI 588 improves TJ protein expression.** Rats were fed a control (CSAA) diet, CDAA diet, or CDAA diet plus MIYAIRI 588 for 8 weeks. MIYAIRI 588 was administered after CDAA diet feeding for 2 weeks. Cont, control. The organization and distribution of occludin protein in intestinal tissues were examined by immunohistochemical staining. Arrows indicate a disrupted intestinal barrier. Data are representative of 6 individual intestinal sections. Scale bars  = 500 µm (upper panels) or 250 µm (lower panels).
(TIF)

Figure S4 **Expression of Nrf2 was induced by MIYAIRI 588 treatment in the liver.** Rats were fed a control (CSAA) diet, CDAA diet, or CDAA diet plus MIYAIRI 588 for 16 weeks. MIYAIRI 588 was administered after CDAA diet feeding for 2 weeks. Cont, control. Nrf2 expression in the liver tissue sections was evaluated by immunostaining. Data are representative of 6 individual liver sections. Original magnification, ×40.
(TIF)

Figure S5 **Expression of Nrf2 was detected in the intestinal tissues by MIYAIRI 588.** Rats were fed a control (CSAA) diet, CDAA diet, or CDAA diet plus MIYAIRI 588 for 8 weeks. MIYAIRI 588 was administered after CDAA diet feeding for 2 weeks. Cont, control. Nrf2 expression in the intestinal tissues was assessed by western blot analysis.β-actin expression was analyzed as a loading control.
(TIF)

Figure S6 **MIYAIRI 588 improves expression of SIRT1 in the liver.** Rats were fed a control (CSAA) diet, CDAA diet, or CDAA diet plus MIYAIRI 588 for 8 weeks. MIYAIRI 588 was administered after CDAA diet feeding for 2 weeks. Cont, control. Hepatic SIRT1 expression was examined by western blot analysis under regular feed conditions or fasted conditions. α-tubulin expression was analyzed as a loading control.
(TIF)

Figure S7 **MIYAIRI 588 prevents the progression of CDAA diet-induced liver fibrosis and cirrhosis.** Male Fischer 344 rats were fed a CSAA diet (Cont), CDAA diet, or CDAA diet plus MIYAIRI 588 for 8 or 16 weeks. MIYAIRI 588 was administered after CDAA diet feeding for 2 weeks. The extent of hepatic fibrosis was assessed by Sirius-red staining. Data are representative of 6 individual liver sections. Original magnification, ×40. The fibrosis area was assessed using image analysis techniques for calculating the ratio of connective tissue to the whole area of liver sections stained with Sirius-red. Data are expressed as means ± SD. ** p < 0.01 compared with the CDAA diet-fed group.
(TIF)

Figure S8 **Nrf2 expression presented in HSCs of liver.** Rats were fed a CDAA diet plus MIYAIRI 588 for 8 weeks. MIYAIRI 588 was administered after CDAA diet feeding for 2 weeks. Double immunofluorescence staining showed the localization of desmin (green, upper left panel) and Nrf2 (red, upper middle panel) in the liver sections. A merged image of desmin and Nrf2 staining is presented in the upper right panel. Likewise, the localization of α-SMA (green, lower left panel) and Nrf2 (red, lower middle panel) in the liver sections are shown. A merged image of α-SMA and Nrf2 staining is presented in the lower right panel. Nuclei (blue) were stained with TOTO-3. Data are representative of 6 individual liver sections. Scale bars  = 20 µm.
(TIF)

Figure S9 **Distinct staining patterns of Nrf2 within the GST-P positive area in liver.** Rats were fed a CDAA diet or CDAA diet plus MIYAIRI 588 for 16 weeks. MIYAIRI 588 was administered after CDAA diet feeding for 2 weeks. Double immunofluorescence staining showed the localization of GST-P (green) and Nrf2 (red) in the liver sections. A similar size of GST-P positive foci in the tissues sections was investigated both in the CDAA diet and the CDAA diet plus MIYAIRI 588 groups. A merged image of GST-P and Nrf2 staining is presented in the right column. Nuclei (blue) were stained with TOTO-3. Data are representative of 6 individual liver sections. Scale bars  = 20 µm.
(TIF)
