# Supplementary figures and images for: Butyrate-Producing Probiotics Reduce Nonalcoholic Fatty Liver Disease Progression in Rats: New Insight into the Probiotics for the Gut-Liver Axis
Source: PLoS One. 2013 May 16;8(5):e63388. doi: 10.1371/journal.pone.0063388 (PMC3656030; doi:10.1371/journal.pone.0063388)

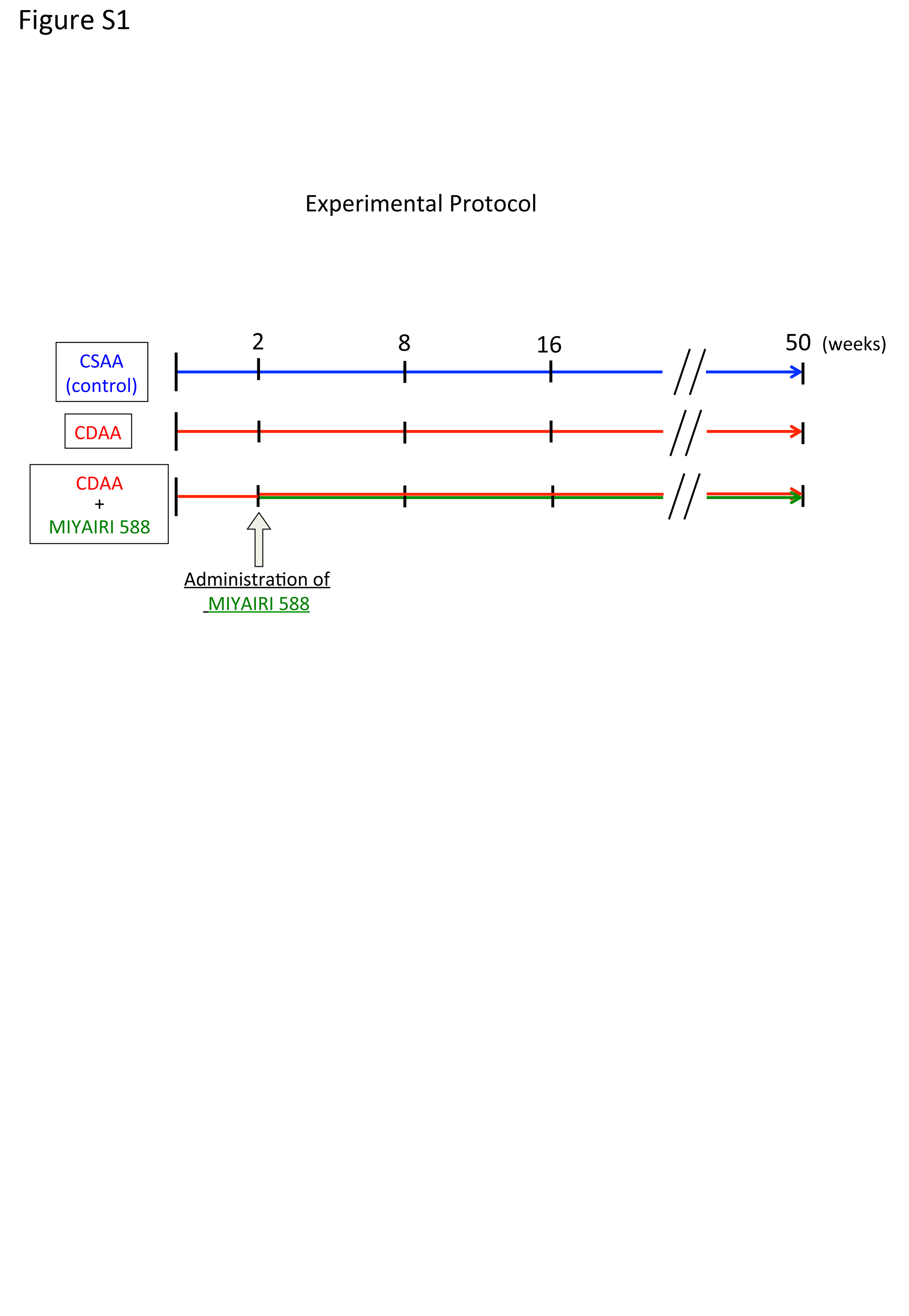

Supplement: Figure S1 — Schematic representation of the experimental protocol. To generate the model of nutrient-induced NASH, male Fischer 344 rats received a CDAA diet. Control group received a corresponding control CSAA diet. In the CDAA plus MIYAIRI 588 group, 10% of the total amount of CDAA diet was replaced with excipients containing MIYAIRI 588 at 2 weeks after the commencement of this experiment. In the both CSAA and CDAA group, 10% of the total amount of the diet was replaced with the same amount of excipients (placebo) only. Rats were killed on 8, 16, and 50 weeks after completion of the diet regimen. (TIF) [file pone.0063388.s001.tif]

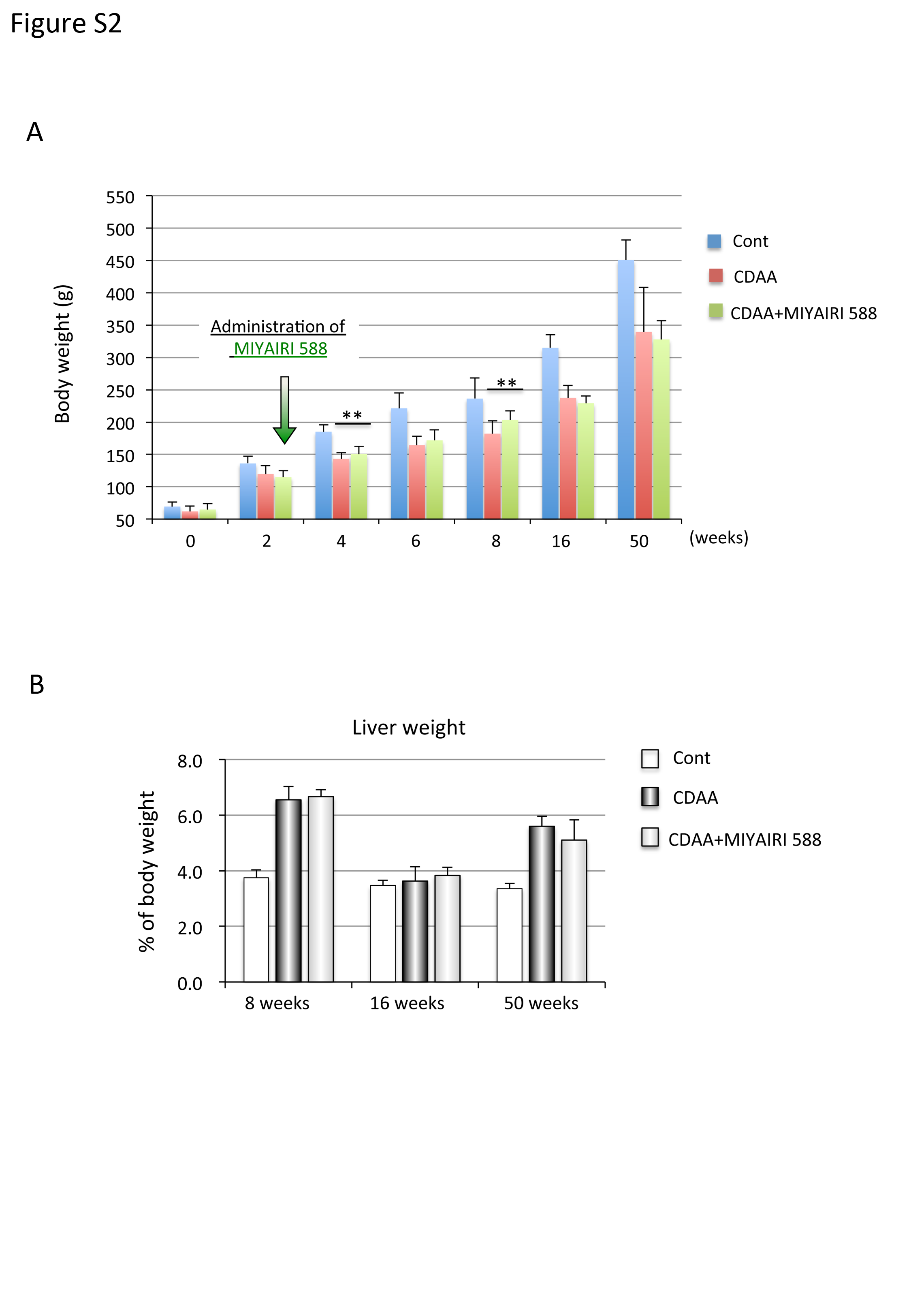

Supplement: Figure S2 — Effects of growth and liver weight by MIYAIRI 588. Cont, control. (A, B) Body weight gain and liver-to-body weight were measured at indicated time points (n = 6–10 per group). Values are expressed as means± SD. **p < 0.01 vs. the CDAA diet-fed group. (TIF) [file pone.0063388.s002.tif]

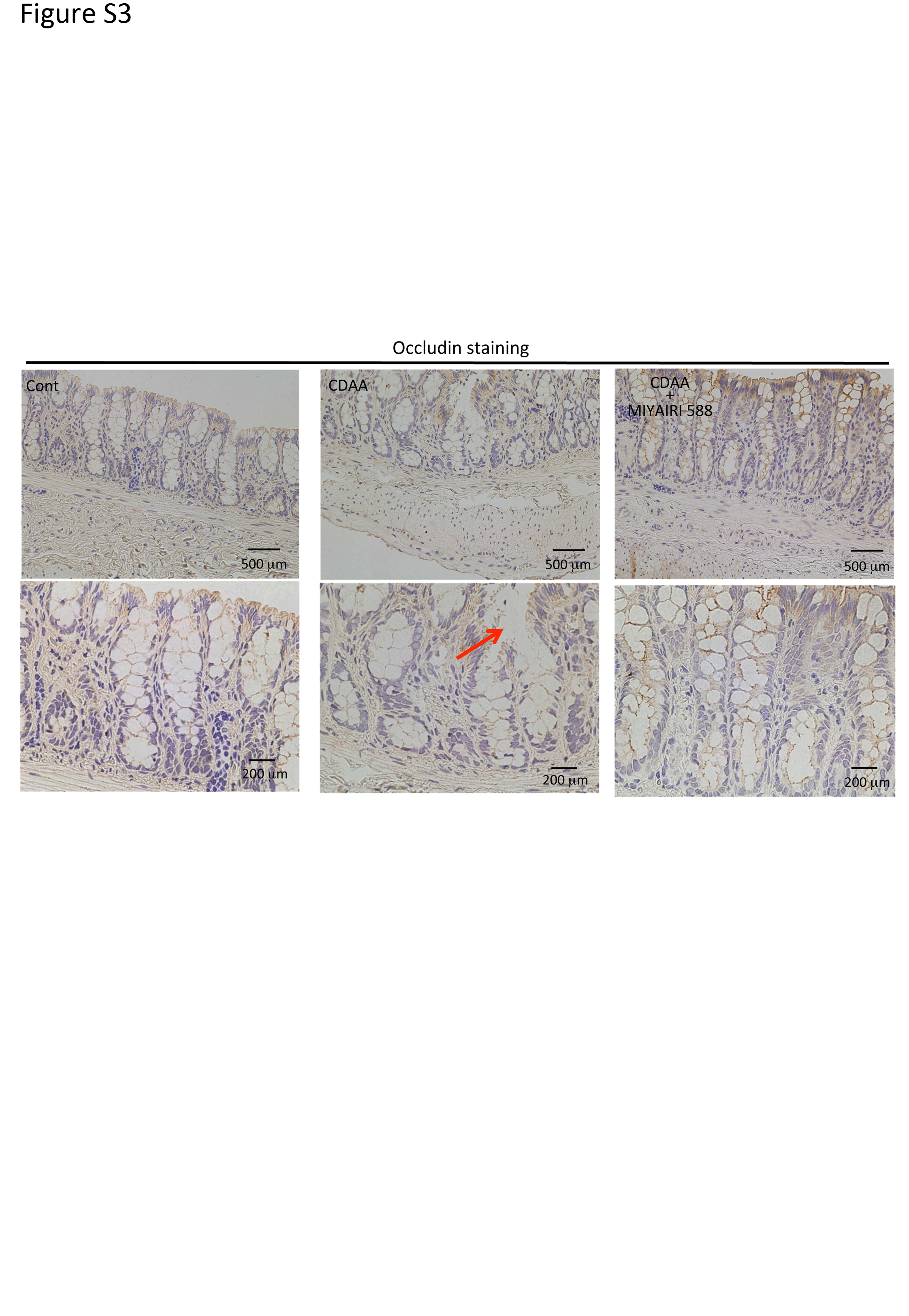

Supplement: Figure S3 — MIYAIRI 588 improves TJ protein expression. Rats were fed a control (CSAA) diet, CDAA diet, or CDAA diet plus MIYAIRI 588 for 8 weeks. MIYAIRI 588 was administered after CDAA diet feeding for 2 weeks. Cont, control. The organization and distribution of occludin protein in intestinal tissues were examined by immunohistochemical staining. Arrows indicate a disrupted intestinal barrier. Data are representative of 6 individual intestinal sections. Scale bars = 500 µm (upper panels) or 250 µm (lower panels). (TIF) [file pone.0063388.s003.tif]

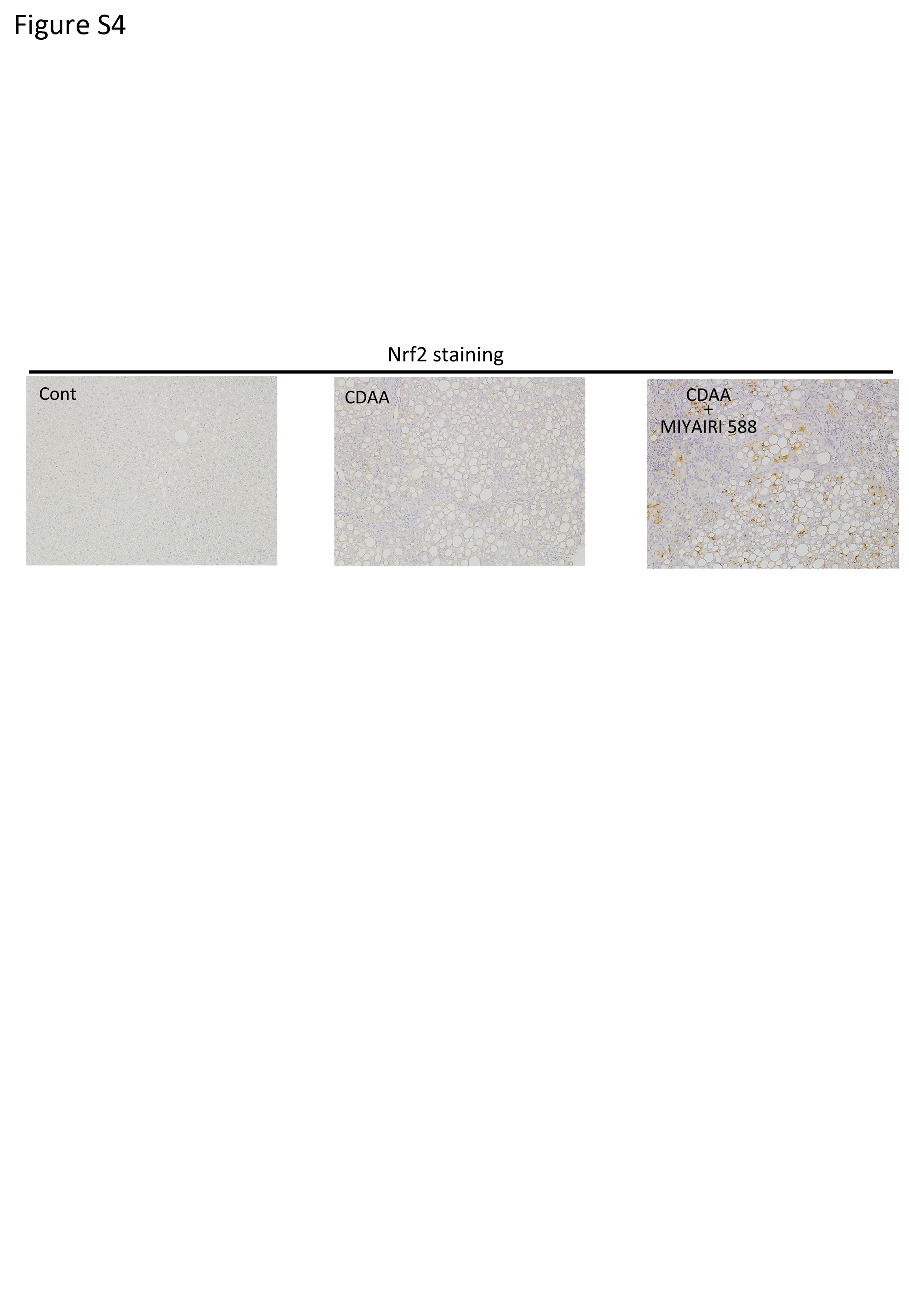

Supplement: Figure S4 — Expression of Nrf2 was induced by MIYAIRI 588 treatment in the liver. Rats were fed a control (CSAA) diet, CDAA diet, or CDAA diet plus MIYAIRI 588 for 16 weeks. MIYAIRI 588 was administered after CDAA diet feeding for 2 weeks. Cont, control. Nrf2 expression in the liver tissue sections was evaluated by immunostaining. Data are representative of 6 individual liver sections. Original magnification, ×40. (TIF) [file pone.0063388.s004.tif]

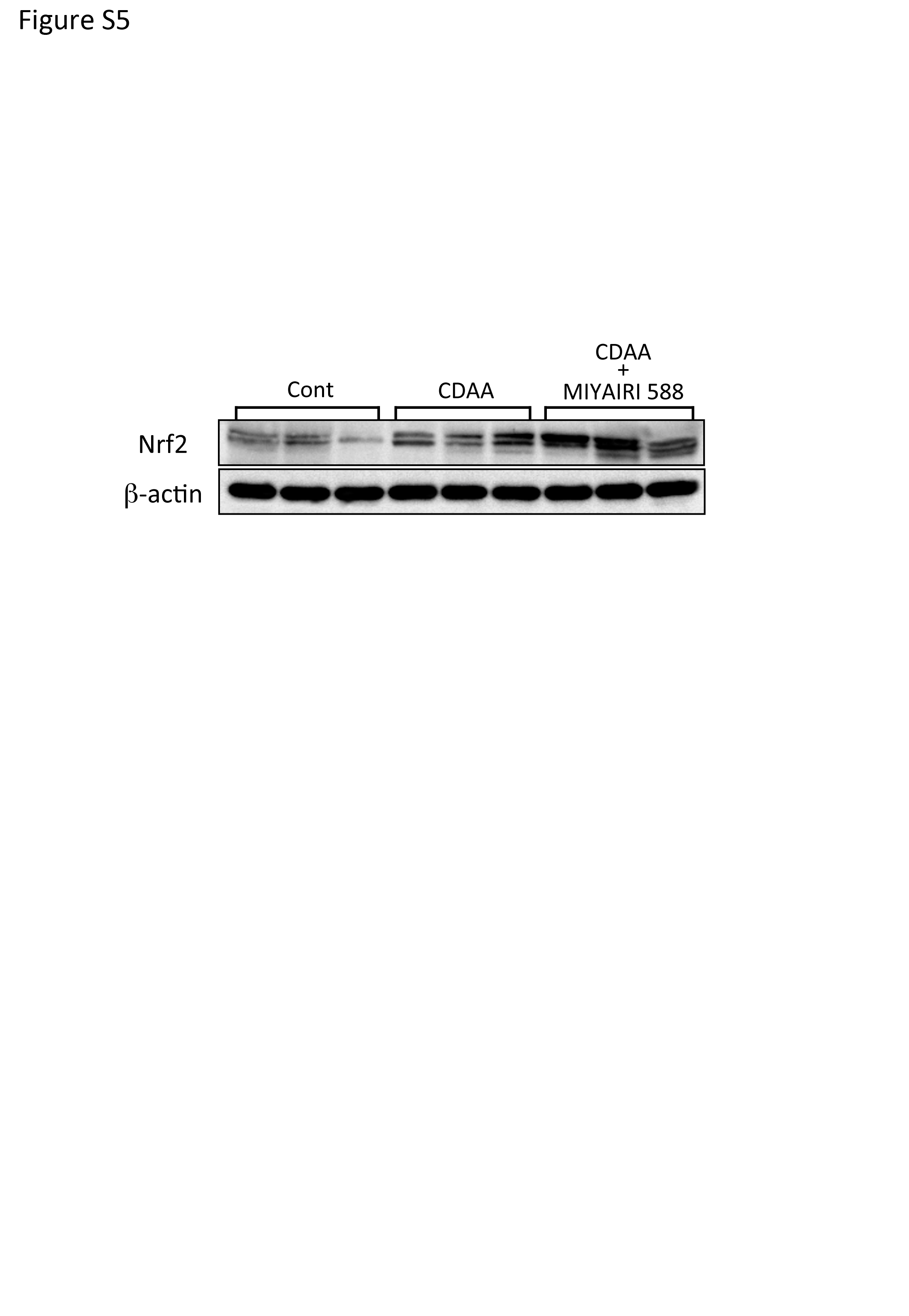

Supplement: Figure S5 — Expression of Nrf2 was detected in the intestinal tissues by MIYAIRI 588. Rats were fed a control (CSAA) diet, CDAA diet, or CDAA diet plus MIYAIRI 588 for 8 weeks. MIYAIRI 588 was administered after CDAA diet feeding for 2 weeks. Cont, control. Nrf2 expression in the intestinal tissues was assessed by western blot analysis.β-actin expression was analyzed as a loading control. (TIF) [file pone.0063388.s005.tif]

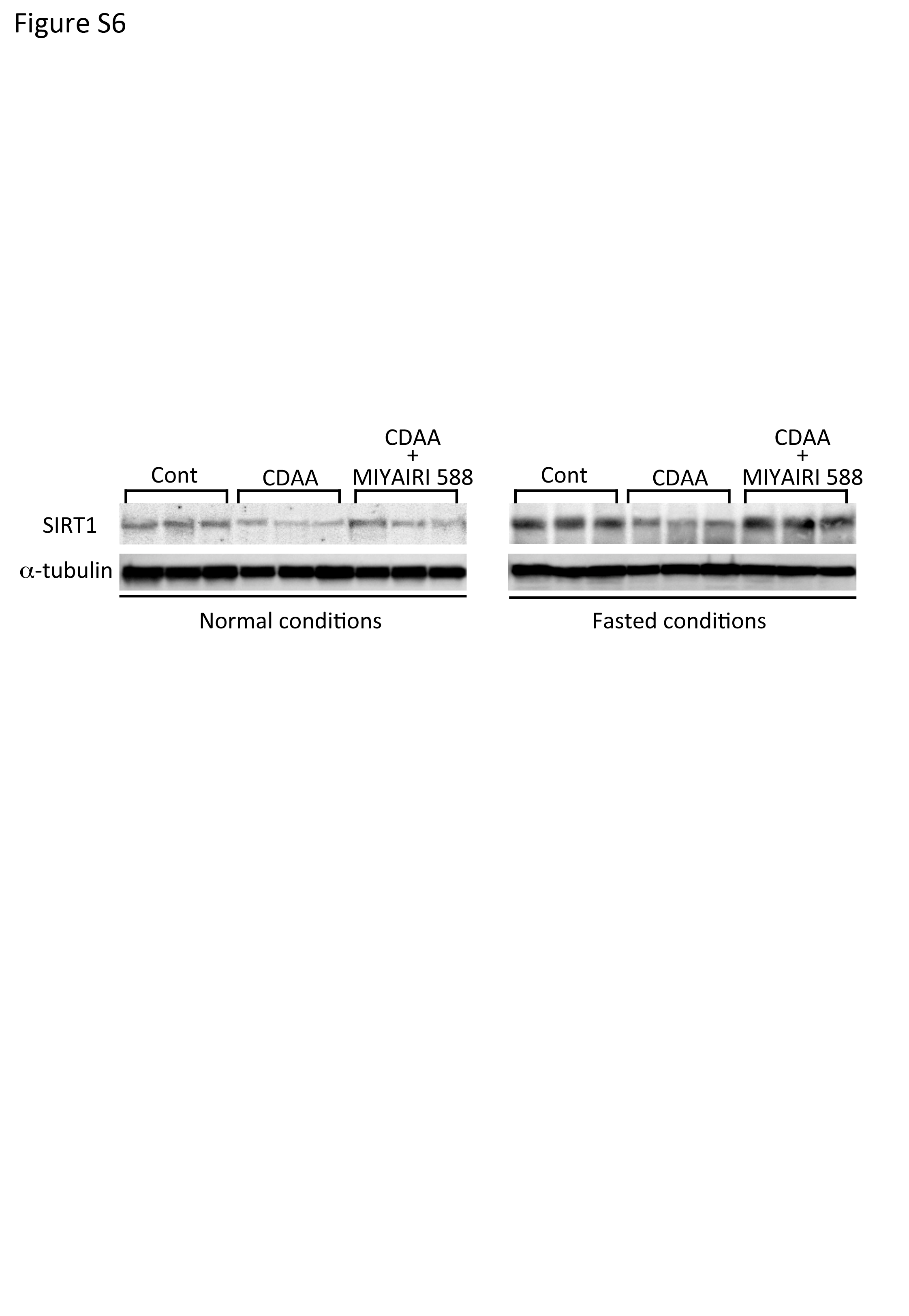

Supplement: Figure S6 — MIYAIRI 588 improves expression of SIRT1 in the liver. Rats were fed a control (CSAA) diet, CDAA diet, or CDAA diet plus MIYAIRI 588 for 8 weeks. MIYAIRI 588 was administered after CDAA diet feeding for 2 weeks. Cont, control. Hepatic SIRT1 expression was examined by western blot analysis under regular feed conditions or fasted conditions. α-tubulin expression was analyzed as a loading control. (TIF) [file pone.0063388.s006.tif]

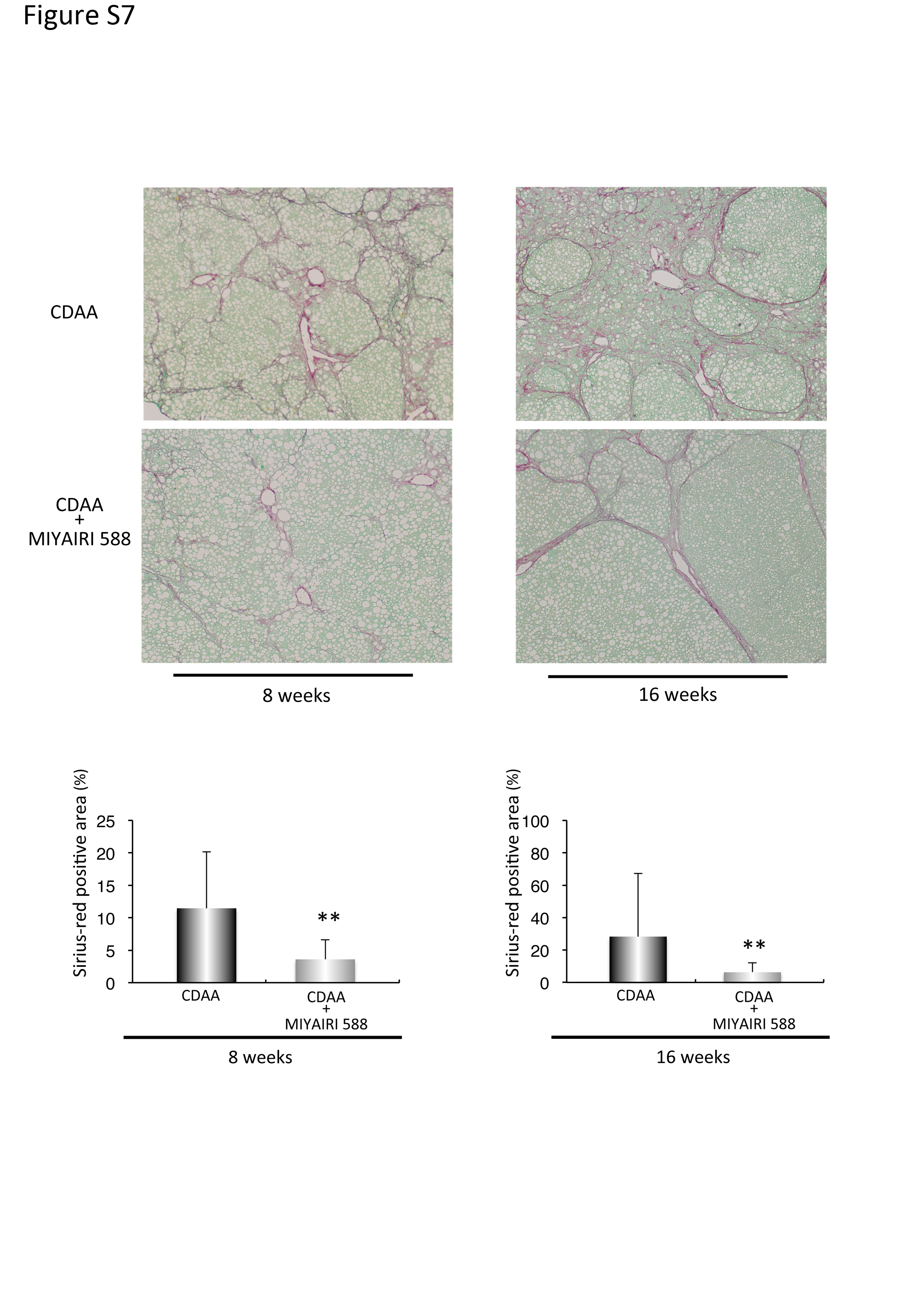

Supplement: Figure S7 — MIYAIRI 588 prevents the progression of CDAA diet-induced liver fibrosis and cirrhosis. Male Fischer 344 rats were fed a CSAA diet (Cont), CDAA diet, or CDAA diet plus MIYAIRI 588 for 8 or 16 weeks. MIYAIRI 588 was administered after CDAA diet feeding for 2 weeks. The extent of hepatic fibrosis was assessed by Sirius-red staining. Data are representative of 6 individual liver sections. Original magnification, ×40. The fibrosis area was assessed using image analysis techniques for calculating the ratio of connective tissue to the whole area of liver sections stained with Sirius-red. Data are expressed as means ± SD. ** p < 0.01 compared with the CDAA diet-fed group. (TIF) [file pone.0063388.s007.tif]

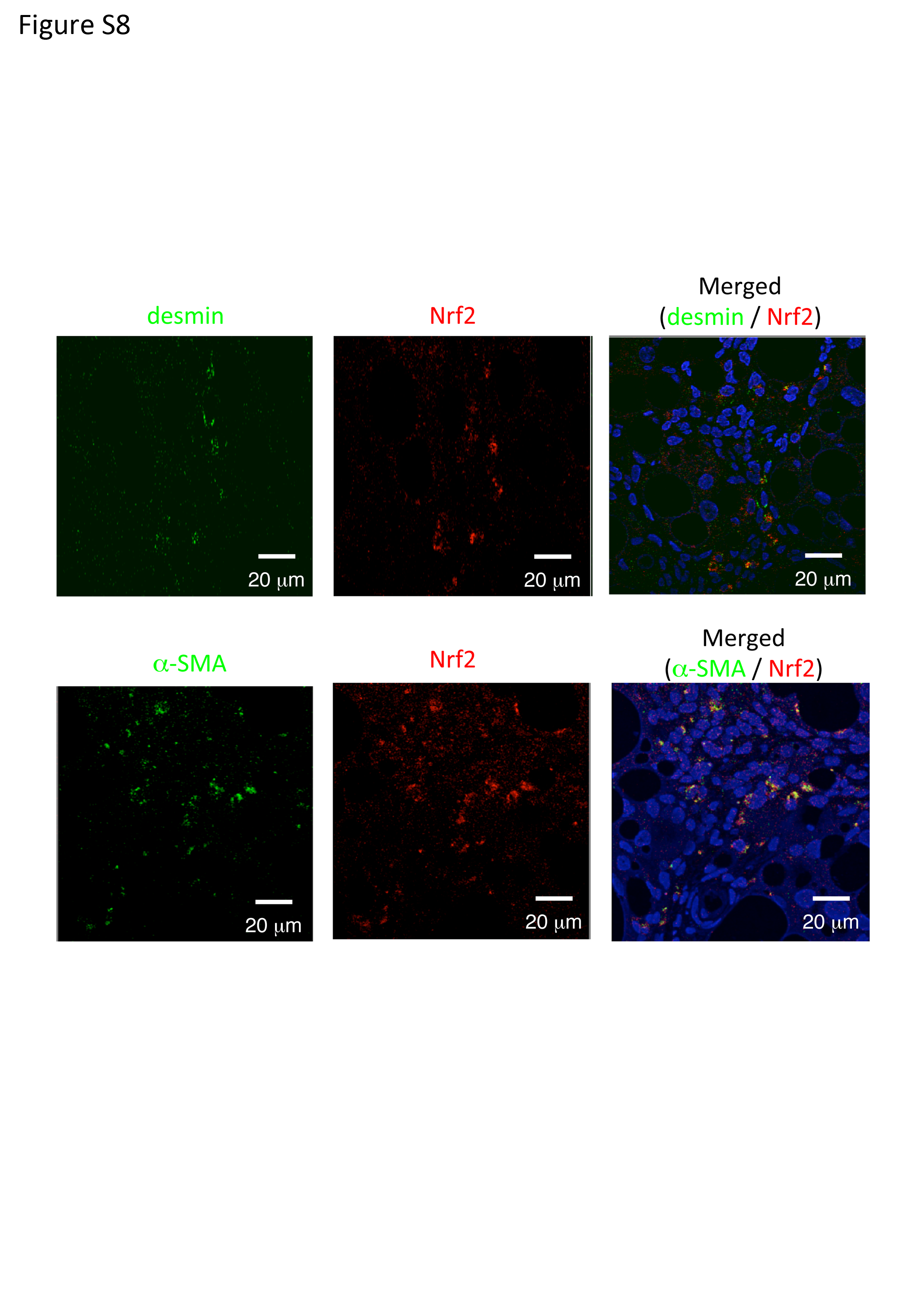

Supplement: Figure S8 — Nrf2 expression presented in HSCs of liver. Rats were fed a CDAA diet plus MIYAIRI 588 for 8 weeks. MIYAIRI 588 was administered after CDAA diet feeding for 2 weeks. Double immunofluorescence staining showed the localization of desmin (green, upper left panel) and Nrf2 (red, upper middle panel) in the liver sections. A merged image of desmin and Nrf2 staining is presented in the upper right panel. Likewise, the localization of α-SMA (green, lower left panel) and Nrf2 (red, lower middle panel) in the liver sections are shown. A merged image of α-SMA and Nrf2 staining is presented in the lower right panel. Nuclei (blue) were stained with TOTO-3. Data are representative of 6 individual liver sections. Scale bars = 20 µm. (TIF) [file pone.0063388.s008.tif]

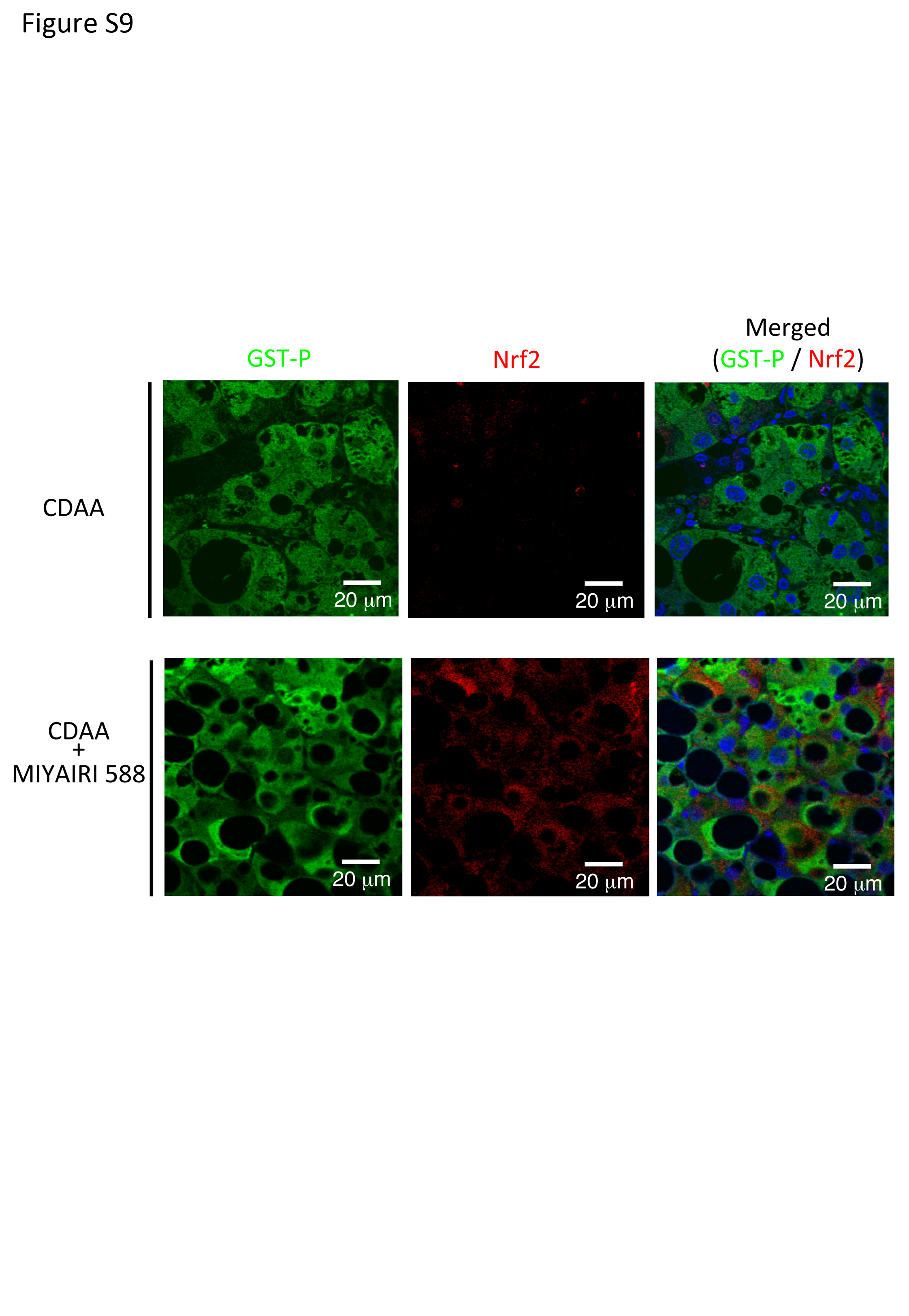

Supplement: Figure S9 — Distinct staining patterns of Nrf2 within the GST-P positive area in liver. Rats were fed a CDAA diet or CDAA diet plus MIYAIRI 588 for 16 weeks. MIYAIRI 588 was administered after CDAA diet feeding for 2 weeks. Double immunofluorescence staining showed the localization of GST-P (green) and Nrf2 (red) in the liver sections. A similar size of GST-P positive foci in the tissues sections was investigated both in the CDAA diet and the CDAA diet plus MIYAIRI 588 groups. A merged image of GST-P and Nrf2 staining is presented in the right column. Nuclei (blue) were stained with TOTO-3. Data are representative of 6 individual liver sections. Scale bars = 20 µm. (TIF) [file pone.0063388.s009.tif]
